# Identification of Dysregulated Genes for Late-Onset Alzheimer’s Disease Using Gene Expression Data in Brain

**Published:** 2020-10-23

**Authors:** Nibal Arzouni, Will Matloff, Lu Zhao, Kaida Ning, Arthur W Toga

**Affiliations:** 1 USC Stevens Neuroimaging and Informatics Institute, Keck School of Medicine of University of Southern California, USA; 2 Computational Biology and Bioinformatics Program, University of Southern California, USA; 3 Neuroscience Graduate Program, University of Southern California, USA

**Keywords:** Late-onset Alzheimer’s disease, Gene expression, Linear mixed models, Brain, AD biomarkers, Biological pathways, Machine learning, Classification

## Abstract

**Background:**

Alzheimer’s Disease (AD) is a neurodegenerative complex brain disease that represents a public health concern. AD is considered the fifth leading cause of death in Americans who are older than 65 years which prioritizes the importance of understanding the etiology of AD in its early stages before the onset of symptoms. This study attempted to further understand Alzheimer’s disease (AD) etiology by investigating the dysregulated genes using gene expression data from multiple brain regions.

**Methods:**

A linear mixed-effects model for differential gene expression analysis was used in a sample of 15 AD and 30 control subjects, each with data from four different brain regions, in order to deal with the hierarchical multilevel data. Post-hoc Gene Ontology and pathway enrichment analyses provided insights on the biological implications in AD progression. Supervised machine learning algorithms were used to assess the discriminative power of the top 10 candidate genes in distinguishing between the two groups.

**Results:**

Enrichment analyses revealed biological processes and pathways that are related to structural constituents and organization of the axons and synapses. These biological processes and pathways imply dysfunctional axon and synaptic transmission between neuronal cells in AD. Random Forest classification algorithm gave the best accuracy on the test data with F1-score of 0.88.

**Conclusion:**

The differentially expressed genes were associated with axon and synaptic transmissions which affect the neuronal connectivity in cognitive systems involved in AD pathophysiology. These genes may open ways to explore new effective treatments and early diagnosis before the onset of clinical symptoms.

## Introduction

Alzheimer’s disease (AD) is a progressive neurodegenerative brain disorder which is a severe detriment to functional and cognitive abilities. The symptomatic cognitive decline and impairments usually appear at the age of 65 or older. However, this age-related sporadic Late-Onset of AD (LOAD) is not the only form. Early-Onset familial Alzheimer’s disease (FAD) is a rare form of AD with symptoms developing in people in early ages that can occur in their 30s or 40s. Normal aging is not typically associated with dementia and AD symptoms, but the risk of developing AD increases with aging.

The number of deaths from Alzheimer’s dementia increased by 89% between 2000 and 2014. AD is considered the sixth leading cause of death in the United States and the fifth leading cause of death in Americans who are older than 65 years [1,2]. The number of Americans living with AD is projected to increase to 13.8 million by 2050 which prioritizes the importance of understanding the etiology of AD in its early stages before the onset of symptoms. The pathogenesis of Alzheimer’s disease is complex and not fully understood with causes that include lifestyle and environmental factors besides genetics. Although it is more difficult to characterize and validate the effects of non-genetic factors in the progression of AD, their whole contribution as an AD risk is almost definite [3,4]. The genetic studies on the etiology of AD have been successful in identifying genes with universal acceptance to their contributions in AD progression. The early-onset FAD is believed to be passed entirely through genetics and caused by mutations in genes like Presenilin 1 (PSEN1), Presenilin 2 (PSEN2) and Amyloid-β precursor protein (AβPP) that are mainly involved in the formation of amyloid-β proteins [5–8]. In both LOAD and FAD, the misfolded beta-amyloid peptides mixed with other proteins and fragments of nerve cells can cause the formation of amyloid plaques which affect the cortical and deep brain tissues [9–11]. Apolipoprotein E (APOE) allele 4 is also established as a high genetic risk for sporadic late-onset AD besides being reported as a risk factor for cardiovascular disease [7,12–14].

Neurofibrillary Tangles (NFTs) are also related to AD progression caused by abnormal tau proteins. The main constituent of NFTs is tau protein which is encoded by the gene Microtubule associated protein tau (MAPT). Abnormal tau phosphorylation is believed to be caused by oxidative stress and iron [15]. NFTs initially affect the entorhinal cortex and then spread to the neocortex and hippocampus of the brain [16]. It is hypothesized that the neurofibrillary tangles of AD brains are most likely formed and deposited after the initial formation of amyloid plaques which can induce the formation of NFTs [17,18]. Most previous research studies on AD genetics have used Genome wide association studies (GWAS) techniques besides genetic linkage and gene expression studies.

Gene expression data has been used to detect AD biomarkers and investigate the biological processes and molecular functions that are involved in AD progression. Gene expression profiling allows genome wide measurements of transcriptomic data. This type of data analysis can give insight into relating AD and its clinical symptoms with gene interactions that play an essential role in AD development. Recent advances in the scale of gene expression data and genomics have allowed the collection of genomic data from multiple tissues and brain regions of the same individual [19]. Since AD progression is sequential and affects many brain regions, it is crucial to integrate gene expression information from multiple brain regions that are part of the cognitive system in order to determine the degree to which the cognitive system is affected [20]. Several research studies found dysregulated levels of gene expression in both grey and white matters involved in AD [21,22].

In this study, new dysregulated genes as AD biomarker candidates were identified using a linear mixed model for differential expression analysis with repeated measures by integrating gene expression data from multiple brain regions. Gene ontology and pathway enrichment analyses were also conducted on the significant differentially expressed genes to provide more information on their biological implications. Machine learning classification techniques were used on the top candidate genes to reveal the discriminative power in distinguishing AD and control samples. The dysregulated genes in this study can be defined as the AD associated genes which are differentially expressed and reflect statistically significant transcriptional changes in the brain of AD subjects compared to healthy controls. These AD associated genes and their underlying pathways can help further understand the pathogenesis of the disease. The identified genes definitely contribute to AD progression and development and may open ways to explore new effective treatments at early stages before the onset of clinical symptoms especially that AD has limited dementia at early stages which can result in misleading diagnosis of the disease. The data used in this analysis was previously published in Miller et al. [23]. However, the authors did not consider the integration of the gene expression data from all four brain regions and their aggregate results. The novelty of the study lies in the integration of multiple brain regions per subject in the analysis using linear mixed model to resolve the correlation structure in contrary to previous research studies which mainly focused on specific brain regions. The novelty of the study also lies in the new differentially expressed genes that were found and not previously reported as AD associated genes.

## Materials and Methods

### Data and pre-processing

RNA sequencing data was obtained from the Aging, Dementia and Traumatic Brain Injury (TBI) Study via the Allen Brain Institute (http://aging.brain-map.org). This dataset, derived from a subset of the Adult Changes in Thought (ACT) cohort, consists of neuropathologic, molecular and transcriptomic data for the postmortem brains of 55 TBI and 52 matched control subjects [23]. We focused here on the control group without previous TBI. Among the control group, 15 subjects had diagnosed AD, 7 subjects had dementia from other causes and 30 had no dementia. We used these 15 AD and 30 control subjects to investigate the differential gene expression in AD. All subjects have signed a consent form in order to participate in the study. The demographics of these 45 subjects used in the analysis are shown in Table 1. All 15 AD subjects were considered late-onset AD cases with mean age of death of 89.6 years. For each subject, gene expression data was available from four different brain regions: The hippocampus (HIP), the Temporal Cortex (TCx), the Parietal Cortex (PCx) and the Forebrain White Matter (FWM). RNA sequencing data was missing in some regions for some subjects yielding a total number of 165 samples with 111 samples in the control group and 54 samples in the AD group (Table 1).

Additionally, the data was analyzed using only 3 brain regions (HIP+TCx+PCx) with a total number of 124 samples (84 control samples and 40 AD samples). The data was also analyzed using only 2 regions (HIP+TCx) with a total number of 83 samples (57 control samples and 26 AD samples). The percentages of AD subjects and control subjects that have APOE Ɛ4 allele were 33.33% and 10% respectively. Advanced staging of the subjects was not available other than the Braak stages. Table 1 shows the mean of Braak stages for control and AD subjects. The mean Braak stage for AD subjects was around 4 with 6 subjects out of 15 had a Braak stage less than or equal to 4. The mean Braak stage for control subjects was around 3 with 28 subjects out of 30 had a Braak stage less than or equal to 4. The RNA sequencing data, which consisted of expression data for 50,281 genes, was normalized into FPKM values across all samples to account for processing batch and RNA quality. All 50,281 genes were used in the analysis without filtering in order to get a wide range of genomic transcriptional candidates that can be associated with AD.

### Linear Mixed-Effects Model (LMM) for differential gene expression analysis

Multiple samples were collected from different brain regions of the same subject which suggested a repeated measure design for differential gene expression analysis because samples from the same subject are correlated. This violates the common assumption for statistical tests that the samples should be statistically independent. Ignoring the correlation between samples in a repeated measure design will not yield consistent and effective estimates and will not control type I error rate. Linear mixed models allow using both fixed and random effects that deal with the non-independence arising from a hierarchical multilevel structure in the data. In our analysis, individuals were taken as random effects in fitting the linear mixed model. The Limma package was used which is an open source R package available through the Bioconductor project [24–26]. Limma entails many methods that can handle complex experimental designs and overcome the small sample size problem by borrowing information between genes using an empirical Bayes approach and resulting in moderated t-statistic [27].

The Voom method was used that generates precision weights for each observation to account for precision variations between different observations [28]. It estimates a mean-variance trend using Locally Weighted Regression (LOWESS) and it predicts the variance of each observation. The Voom precision weights are the inverse of the predicted observation variance. Squeezing the gene wise variances to the common trended variance will reduce the false positive rate for genes with small variances and improve the detection power for differentially expressed genes with larger variances. The linear mixed model was estimated using the lmer function in the lme4 package [29]. The null hypothesis in LMM usually lies in whether one or more of the regression coefficients as contrast estimators are equal to zero. In this analysis, our interest lies solely in the contrast estimator that corresponds to the disease status to analyze the differential expression between Alzheimer’s disease and healthy control samples. The gene expression data was first adjusted for age and sex and then a Logarithmic transformation was performed on the data to counteract the unequal variability between large and small values [30,31].

### Gene ontology and gene set enrichment analyses

The statistically significant Differentially Expressed Genes (DEGs) with the smallest P-values obtained by using LMM were used with gene ontology enrichment analysis to identify the enriched dysfunctional biological implications associated with these genes. Gene Ontology tool powered by Panther was used in this analysis. The enriched biological processes, molecular functions and cellular components associated with the top ten genes and all differentially expressed genes were identified using Bonferroni correction for multiple testing with a P-value threshold of .05. In addition to Panther, the Database for Annotation, Visualization and Integrated Discovery was used to identify some enriched biological themes and diseases associated with the provided list of the top ten genes [32,34].

Gene set enrichment analysis was performed to find the significantly enriched co-regulated gene sets or pathways. Each pathway represents a certain biological or molecular function of interest. The gene sets are usually defined from external sources like Gene Ontology database or from previously established research studies [30]. In this analysis, Broad institute GSEA was used with the Reactome gene sets derived from the Reactome pathway database [35]. The 50,281 genes were pre-ranked using the moderated t-statistic derived from the linear mixed model for differential expression. All 50,281 pre-ranked genes were then used with GSEA to find the significantly enriched pathways in both control and AD groups using a threshold P-value of .01 and False Discovery Rate (FDR) of .02.

### AD ML binary classification

Supervised Machine Learning (ML) algorithms were used to identify the discriminative power of the top ten differentially expressed genes in distinguishing between the two categories. Support vector machines using both linear and radial basis function kernels were selected for the binary classification in addition to random forest and quadratic Bayes algorithms [36]. The data was divided into a training set and a test set. The training set consisted of approximately 70% of the samples (114 samples) and the remaining 30% were used for testing to test and approximate how the classifiers generalize to unknown data. There was no overlap in the subjects and samples between the training and testing data to avoid any correlation between samples in both sets. The genes were used as features for each sample with all different combinations of N genes (2 ≤ N ≤ 10) out of 10 genes. For every combination of N genes, the combination with the maximum accuracy on the training data was chosen and the corresponding accuracy on the test data was reported on those N genes to avoid data snooping. Precision and recall scores in addition to the F1 score were reported for the testing accuracy that corresponded to the maximum training accuracy over all combinations.

## Results

### Differentially Expressed Genes (DEGs)

**Figure 1:**
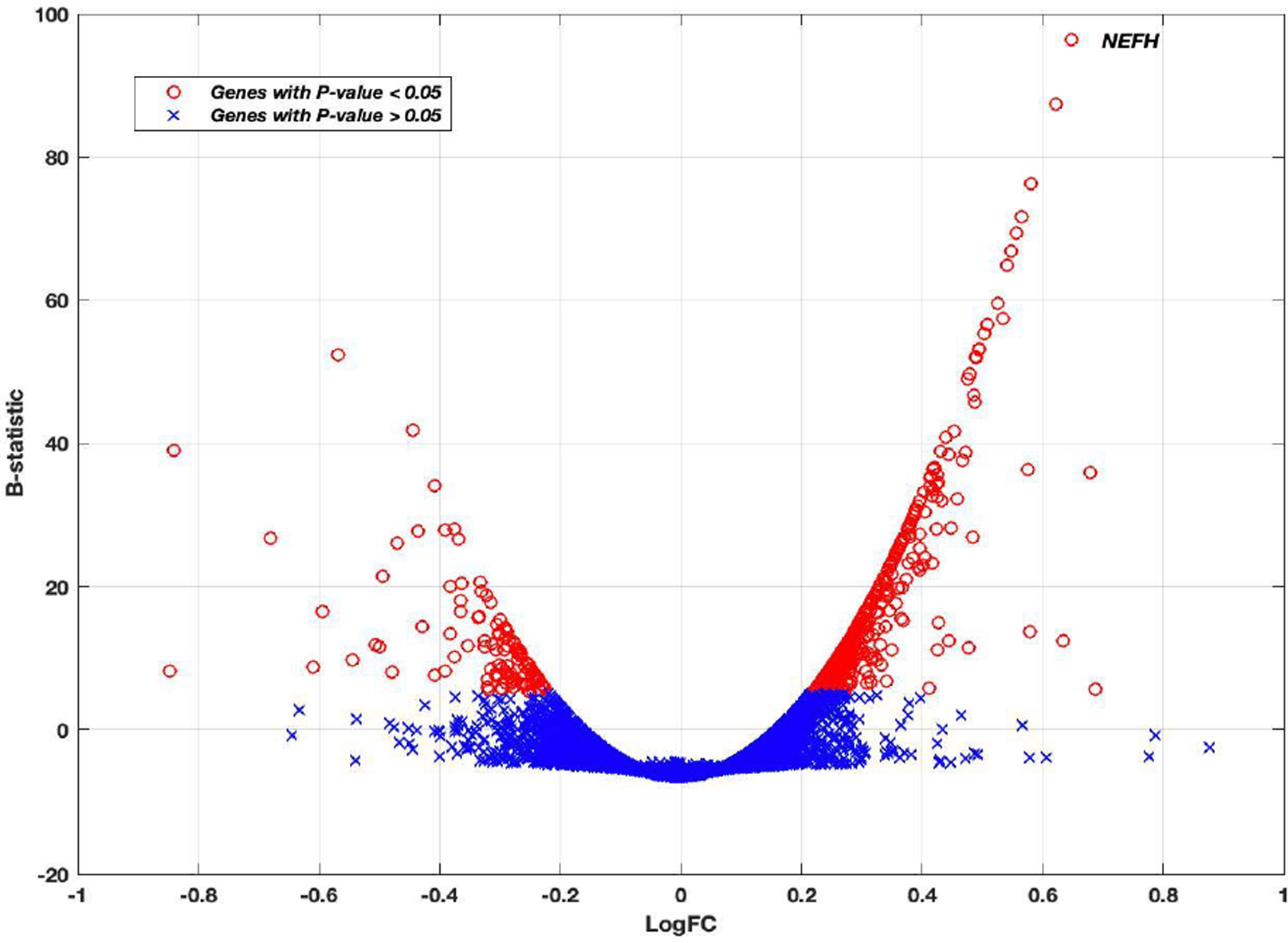
The Volcano plot of Log2 fold change versus B-statistic for all genes. Genes with P-value<.05 are shown as red circles. Otherwise, they are blue crosses. The *NEFH* gene is marked. The *NEFH* gene was the most statistically significant differentially expressed gene.

Using the linear mixed-effects model, the reported P-value associated with each gene was used to assess its differential expression between Alzheimer’s disease and control groups. The final P-values were adjusted for multiple comparison by Bonferroni correction. There were 602 genes out of 50,281 genes that showed significance at a 5% significance level using all four brain regions in the analysis. The results for the top ten genes are shown in Table 2 with columns representing log2 fold change (logFC), average log2 expression (AvExp), moderated t-statistic, corrected P-value and B-statistic for each gene. The B-statistic is the posterior log odds of differential expression derived in [27]. Log fold change was defined in our analysis as AD to control ratio. A positive value indicated that the gene was upregulated in AD. Table 2 also gives more information about the size and location of the top 10 genes in the genome. Supplemental Table 1 contains more information about all 602 DEGs with their statuses in AD and the gene categories. The gene category can be protein coding, RNA gene (non-protein coding) and pseudogene. Among the 602 DEGs, 490 genes were protein coding genes, 74 genes were RNA genes and 29 genes were pseudogenes. The volcano plot in Figure 1 shows how the log2 fold change is changing with the posterior log odds for differential expression. The B-statistic for each gene generally increased with increased significance in differential gene expression. The top 10 differentially expressed genes showed a logFC>0.5 and B-statistic >50. Neurofilament heavy polypeptide (*NEFH*) gene showed the most significant differential expression between Alzheimer’s and control subjects (Figure 1) (Table 2).

**Figure 2:**
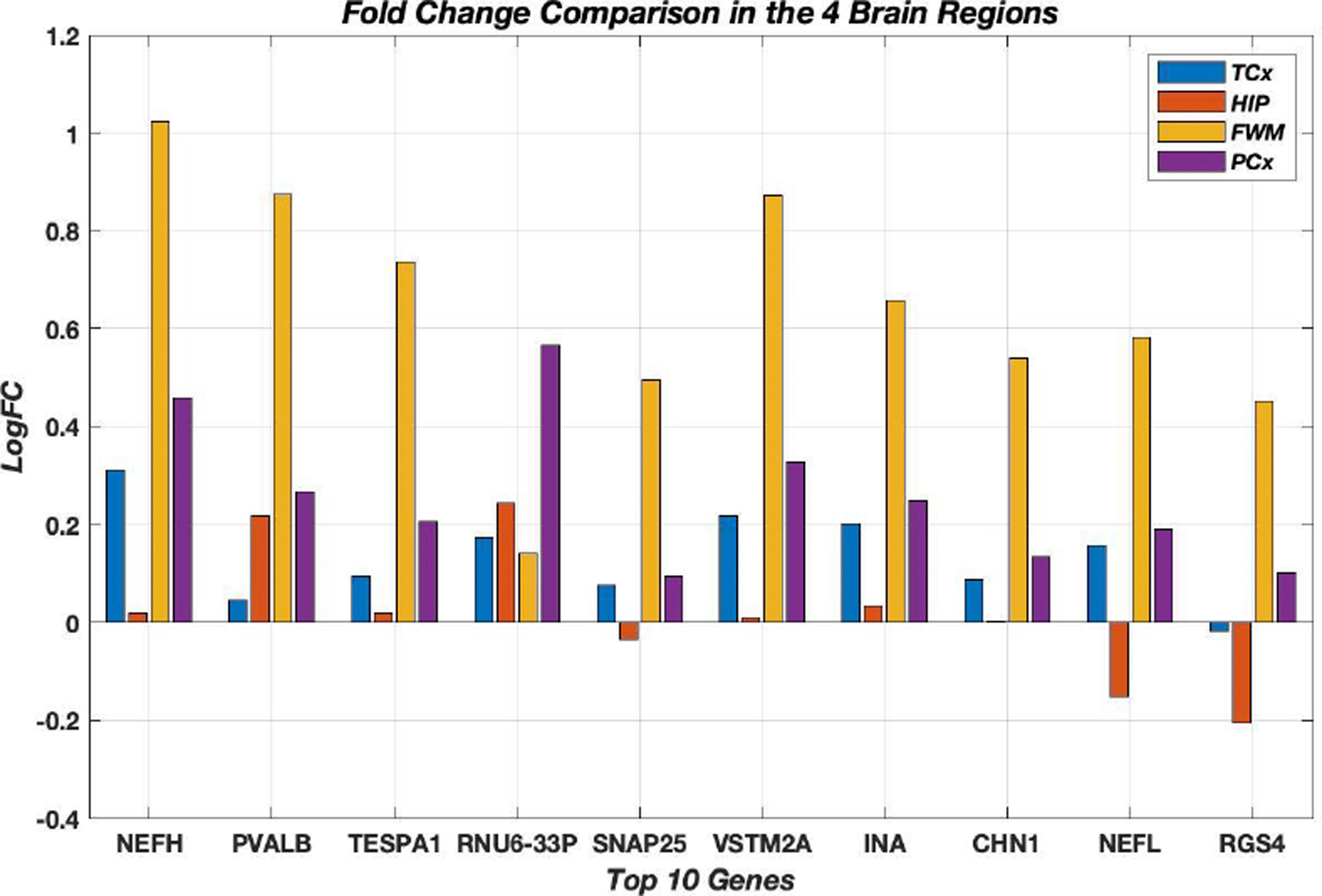
Log2 fold change comparison for the top ten genes in all 4 brain regions. All these genes were up regulated in AD using the four-brain region LMM analysis. All the genes were mostly up regulated in all four brain regions except for *SNAP25, NEFL, RGS4* that were down regulated in the Hippocampus. *RGS4* was also down regulated in the temporal cortex.

Most DEGs, 484 genes out of 602 genes, were upregulated in AD. The top ten differentially expressed genes were all upregulated in AD using all four brain regions in the analysis. Figure 2 shows the comparison of LogFC for the top ten genes in the 4 distinct brain regions. It was noticeable how the LogFC values were high and showed upregulation in FWM for all top ten genes. These top DEGs were mostly dysregulated in the same direction in all four brain regions except for three genes (*SNAP25*, *NEFL*, *RGS4*) that were downregulated in the Hippocampus. The gene *RGS4* was also downregulated in the Temporal cortex. The top ten genes were searched using Agora platform (https://agora.ampadportal.org) which was initially developed by NIA-funded AMP-AD consortium that shares evidence in support of AD target discovery. None of the top ten genes were previously reported to have any RNA expression changes in AD brains and three genes (*NEFL*, *TESPA1* and *SNAP25*) were reported as nominated AD targets according to Agora. The database Alzgene (www.alzgene.org), which is a collection of published AD genetic association studies, was also searched and none of the genes were previously reported in any AD research study according to Alzgene. Few genes out of the top ten DEGs, in particular *NEFL*, *NEFH*, *RGS4* and *SNAP25*, were previously found in research studies to exhibit altered expression levels in various brain cell types in AD brains [37–39] (Figure 2).

The data was also analyzed using (HIP + TCx + PCx) samples excluding FWM samples. Using LMM, there were 146 genes that showed statistical significance in differential expression after Bonferroni correction. There were 90 downregulated genes in AD out of 146 DEGs. Supplemental table 2 contains all the 146 genes and their statuses. There were 90 common genes with the 602 differentially expressed genes using all four brain regions. Fifty-three genes out of 90 common genes were found as downregulated in AD in (HIP+TCx+PCx) analysis. The same 53 common genes were also found to be downregulated in the analysis of four brain regions. Additionally, the data was analyzed using only (HIP+TCx) samples with the accepted hypothesis that AD affects HIP first followed by TCx [40,41]. The differentially expressed genes were only 93 genes. There were 61 downregulated genes in AD out of 93 DEGs. Supplemental Table 3 contains all the 93 DEGs and their statuses. The (HIP+TCX) analysis will have a limited number of samples since there will only be 83 samples in total.

### Enrichment analyses results

The gene ontology analysis was focused on the top ten DEGs as AD biomarker candidates with the most significant differential expression. Gene Ontology database powered by Panther revealed the enriched annotations associated with those genes. The underlying enriched GO terms in biological processes, cellular components and molecular functions were mainly related to neurofilament and postsynaptic cytoskeleton organization and structural constituent of synapses. These GO annotations suggested that the genes are associated with dysfunctional structural brain connectivity in Alzheimer’s disease. Table 3 summarizes the GO terms associated with the top ten genes using a threshold of 0.05 for the Bonferroni corrected P-values. The database for annotation, visualization and integrated discovery clustered the enriched biological themes associated with each gene. All top genes showed that they are mainly expressed in brain tissues. The diseases associated with these genes were shown to be mainly related to neurological and psychological disorders, motor neuron diseases and brain structural connectivity. Gene ontology analysis was also performed on all 602 DEGs and not just the top 10 DEGs. Supplemental Tables 4, 5 and 6 reveal the annotations for biological processes, molecular functions and cellular components respectively. The Gene ontology annotations using all 602 genes gave a more comprehensive list of terms which completely agreed with terms that were found using the top ten genes in table 3. Gene ontology analysis on (HIP+TCx) DEGs did not give any annotations after correction. GO analysis on (HIP+TCx+PCx) DEGs gave few annotations in which “Structural constituent of myelin sheath” was the only annotation in molecular functions that survived after correction and “Neurofilament” was the only annotation in cellular components that survived after correction (Table 3).

**Figure 3:**
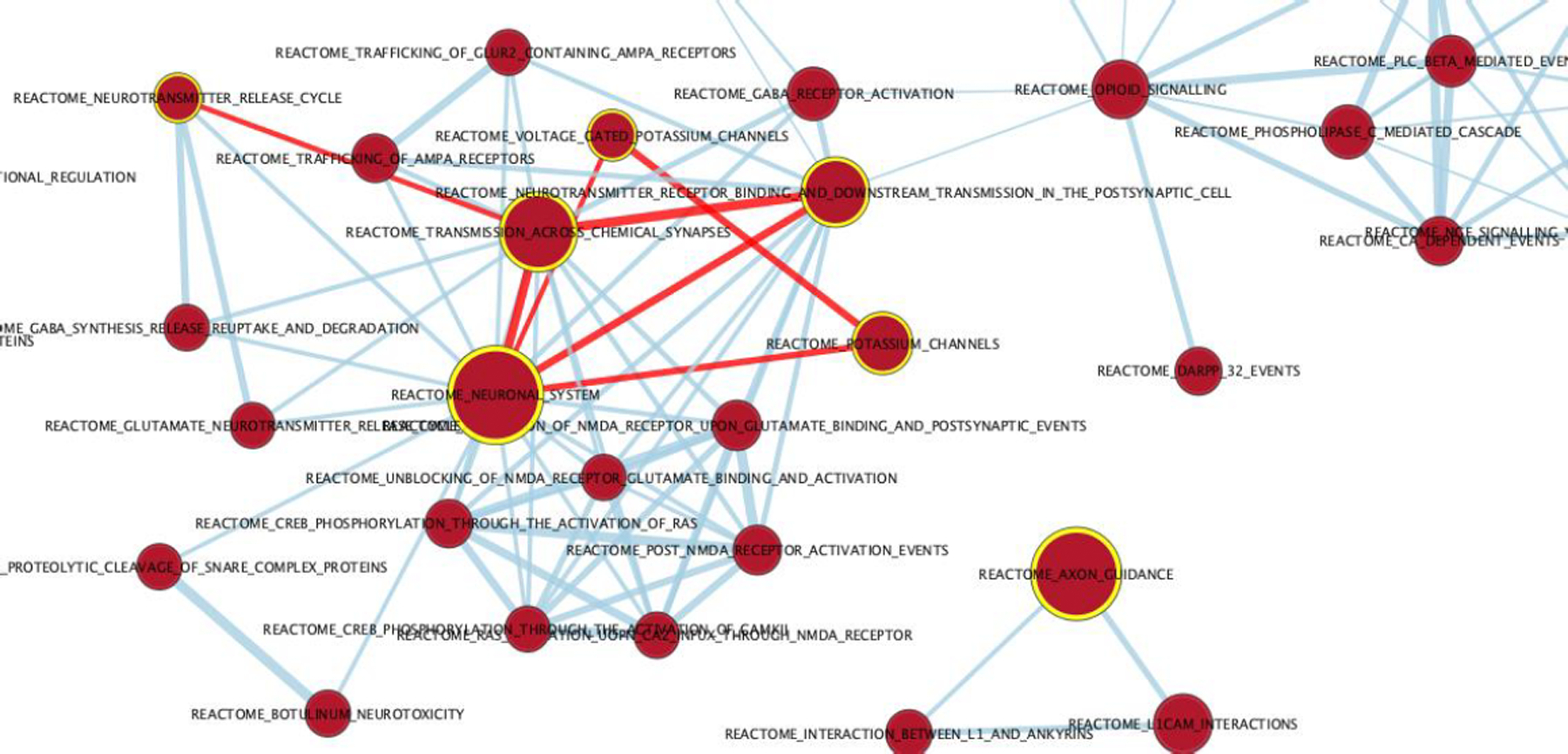
Network graph of the AD enriched pathways. A zoomed-in network graph on the AD nodes representing the up regulated pathways in AD with P-value<.01 and FDR<.02. The yellow highlighted nodes are the most significantly enriched up regulated pathways with red edges showing the connections between those pathways. This is a zoomed-in graph from supplemental figure 1.

Broad institute GSEA was used to reveal the most significant enriched pathways associated with the disease using the Reactome pathways database and using all genes. The pre-ranked genes revealed pathways that were mainly associated with neuronal systems, axon guidance and neurotransmitter release cycle across chemical synapses. The enriched pathway results asserted and agreed with the GO terms and verified that these genes are associated with Alzheimer’s disease. Forty gene sets and forty-nine gene sets were upregulated and downregulated in Alzheimer’s disease respectively with a cutoff P-value of .01 and cutoff FDR of .02. Table 3 shows the top enriched pathways with the resulting enrichment score and gene set size mapped to the 50,281 genes. Cytoscape (3.7.1) was used to visualize the network graphs of the enriched pathways [42]. Supplemental Figure 1 shows the AD upregulated pathways as red nodes and the downregulated pathways as blue nodes. The nodes were clustered separately where there were no connections between the red and the blue nodes. The AD upregulated pathways formed a connected uncomplete graph excluding the axon guidance pathway cluster. Figure 3 shows the top highlighted AD upregulated pathways that are mentioned in table 3 with red edges showing their connections. Neuronal system was the most enriched pathway and it was connected with the transmission across chemical synapses pathway with an edge similarity coefficient of 0.83 since they share 186 genes. The highest similarity coefficient of 0.86 was between the transmission across chemical synapses pathway and neurotransmitter receptor binding and transmission in postsynaptic cell pathway where they share 137 genes. The downregulated pathways in phenotype AD were mainly associated with DNA synthesis, translation and lipoprotein metabolism (Figure 3).

### AD classification results

**Figure 4:**
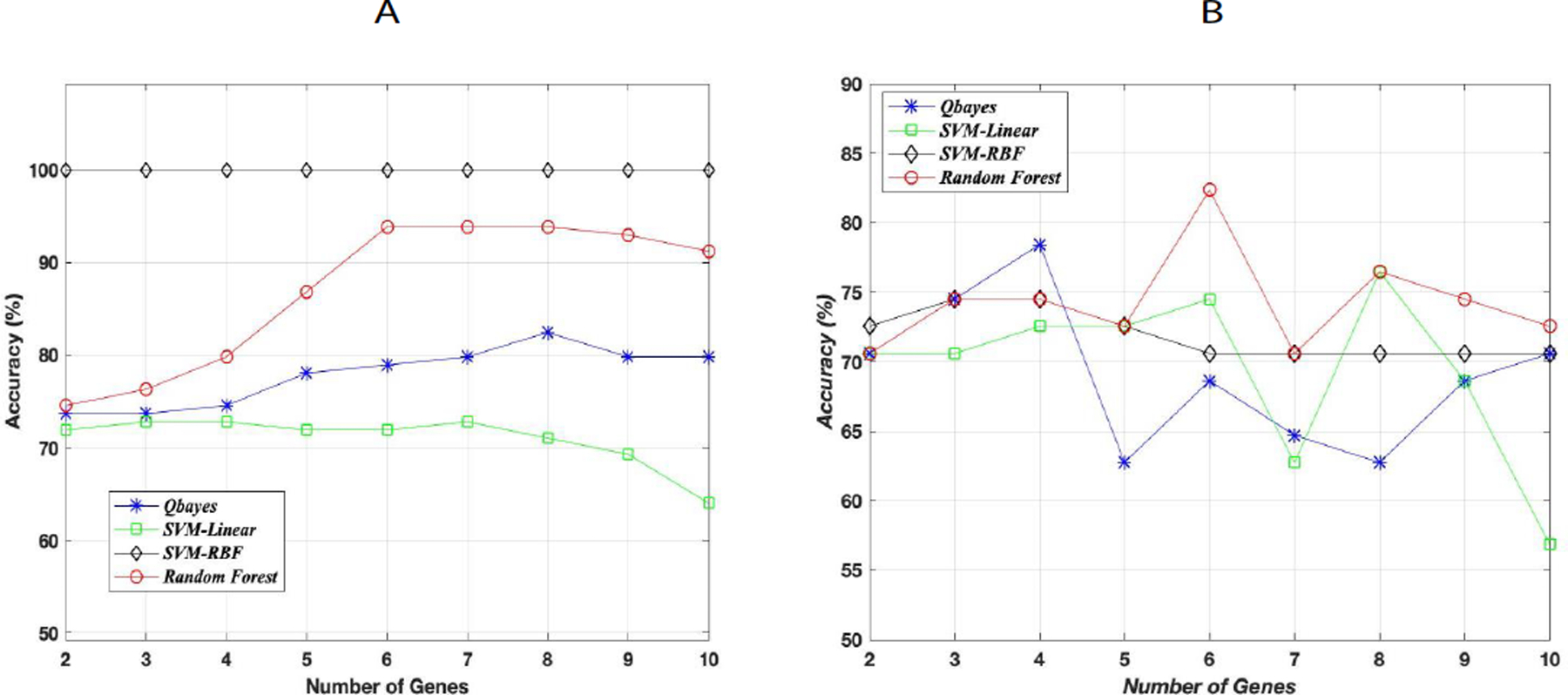
Supervised ML classification using the top ten genes. (A) Classification results on the training data using four algorithms. The plots correspond to the maximum training accuracy achieved on a combination of N genes where 2 ≤ N ≤ 10. (B) Classification results on the testing data. The plots correspond to the testing accuracy on N genes that achieved the highest accuracy on the training data.

Four supervised ML algorithms were used for this binary classification in which the random forest classifier gave the best accuracy on the test data. Figure 4 (A) shows the maximum training accuracy on N genes (2≤N≤10) for all four classifiers. Figure 4 (B) shows the testing accuracy that corresponded to the maximum training accuracy on the same N genes. Although SVM with gaussian kernel gave the highest accuracy on the training data, random forest classifier gave the best generalization accuracy on the test data with an accuracy of 83%. This 83% test accuracy corresponded to the maximum training accuracy on the same six genes. The recall and precision scores for this test accuracy were 0.97 and 0.81 respectively with F1-score of 0.88. The high (100%) accuracy of SVM with gaussian kernel on the training data can be due to overfitting which resulted in a lower accuracy on the test data. The out of sample generalization error has an upper bound ≤ 27% (17% (test error)+10%) with a probability ≥ 0.95 where the 10% is the error bar estimate derived from Hoeffding’s inequality using a tolerance δ = 0.5 (Figure 4).

## Discussion

Gene expression analysis can reveal the biological implications underlying certain diseases. The recent advances in the scale of acquiring genomic data have opened many ways to analyze and explore complex diseases requiring paralleled advances in computational tools and methods. Alzheimer’s disease is a complex disease with etiologies that are not fully understood. In this study, gene expression data was collected from multiple brain regions which are usually affected by Alzheimer’s disease.

It was verified in our analysis using Wilcoxon rank sum test that there were no statistically significant differentially expressed genes between dementia and control samples within the same brain region as it was mentioned in Miller et al. [23]. All four brain regions have been combined in the analysis because AD will affect multiple brain regions, so it is crucial to integrate gene expression information from brain regions which are part of the cognitive system to understand how the cognitive system is affected by AD. Even if these brain regions have distinct anatomical or functional patterns, they are all involved in the development and progression of AD. The genetic and transcriptional AD changes occur concordantly in multiple brain regions that affect the cognitive systems. FWM has distinct cellular content and structure, but several research studies examined and observed similar deregulated levels of gene expression in both grey and white matters involved in AD [22]. Patel et al. reported differentially expressed genes which were common across multiple brain regions in AD using a meta-analysis of AD datasets [21]. They identified some AD specific genes which were expressed consistently in the same direction across multiple brain regions which were both grey and white matter regions. The differentially expressed genes in our analysis using the four brain regions represent AD associated transcriptional changes in most of the brain rather than tissue specific changes limited to one brain region. The used four brain regions cover most of the brain parts. Additionally, modeling multiple brain regions together allows pooling all samples from the data set and consequently increases the statistical power. Sample sizes for post-mortem brain gene-expression studies are typically very small, so being able to combine samples from different regions is advantageous.

The hierarchical multilevel structure of the data can be resolved by using linear mixed models for differential expression. The assumption of independent observations in statistical tests is violated in such hierarchical data. LMMs can be used to resolve this issue with the full use of the data. Other approaches exist in dealing with hierarchical structure, but they don’t take the advantage of using information from all data [43]. Aggregate analysis takes the average of observations from the same subject which can yield consistent results, but it doesn’t consider the advantage of having more observations and may lose important differences by averaging all samples within the same subject. Another alternative is to run separate analysis for each brain region. This separate analysis method can also work, but it will produce many models and again does not take the advantage of having information from other brain regions or subjects simultaneously. Using all available data with LMM will increase the statistical power to detect differentially expressed genes that are AD specific. Ignoring the correlation structure between the samples from all brain regions using Wilcoxon rank sum test did not show any association with neurological disorders or AD. In our study, the top differentially expressed genes produced by LMM integrating all four brain regions showed high association with neurological disorders. The results verified the effectiveness of using LMM on hierarchical structures of gene expression data.

Several previous studies have used post-mortem AD and control samples of different brain regions to investigate differential gene expression in a specific region [23,44]. Typically, these studies examined each region separately in order to investigate the specific changes that take place in each brain region, which can dissect the pathology and impact over a brain region. The main purpose of our study was to analyze AD pathology in most of the brain and not to study distinct functions or transcriptional patterns of different and specific brain regions. Some studies attempted to integrate all brain regions, but they did not use a single appropriate statistical method that integrate all brain regions simultaneously. Wang et al. found the differentially expressed genes in each of six brain regions [44]. In an attempt to integrate all data, they combined the differentially expressed genes from all regions by taking the union and formed a differential co-expression network representing all brain regions without using a statistical test that combines all regions.

The genes *APOE*, *AβPP*, *PSEN1* and *PSEN2* did not show any significant differential expression in our analysis after correction for multiple comparisons using Bonferroni adjustment. Some previously reported AD associated genes showed differential expression in this study. For example, the gene *RGS6* was found differentially expressed in our analysis of using four brain regions. The gene *RGS6* was previously reported as an AD associated gene [45]. The gene *FIBCD1* was also found to be differentially expressed. The gene *FIBCD1* encodes for Fibrinogen C Domain-Containing protein 1 which was previously reported as associated with CNS inflammation, cognitive decline and inhibition of repair [46]. Some previously reported AD biomarkers were not found to be among the 602 DEGS. For example, in addition to the genes *APOE*, *AβPP*, *PSEN1* and *PSEN2*, the gene *A2M* which encodes alpha-2-macroglobulin was reported as an AD biomarker, but it was not found as differentially expressed in this study [47–49]. Similarly, the gene *BDNF* which encodes brain-derived neurotrophic factor protein was previously reported as an AD biomarker and it was not among the DEGs [50]. Few genes out of the top ten DEGs, in particular *NEFL*, *NEFH*, *RGS4* and *SNAP25*, were previously found to exhibit altered expression levels in various brain cell types in AD brains [37–39].

A more specific examination of the top ten genes showed that those top DEGS can be highly linked to AD. The gene *NEFH* encodes the heavy neurofilament protein and the gene *NEFL* encodes the light chain neurofilament protein. The gene *INA* encodes internexin neuronal intermediate filament protein alpha. Neurofilaments play an important role in axonal growth and intracellular transport to axons and dendrites. The number of neurofilaments in the axon increases as the axon becomes myelinated, mature and connected to its target neuronal cell. The increase in the neurofilament number determines the cross-sectional diameter of the axon and its transport. Any abnormalities in the organization or structural constituents of the neurofilaments or synapses can lead to abnormal axon or synaptic transmission and can signify a dysfunctional connectivity between neuronal brain cells. It has been reported in previous research studies that neurofilament mutations and post-translational modifications can lead to Alzheimer’s disease and neurological diseases [51,52]. The gene *PVALB* encodes a high affinity calcium ion-binding protein that is structurally and functionally similar to calmodulin and troponin C. Calcium binding proteins like Calmodulin have been previously reported to be involved in Amyloid plaques formation and are linked to Alzheimer’s disease [53]. *TESPA1* is also a protein coding gene that codes for thymocyte expressed positive selection associated 1. This gene was nominated by Agora as an AD target since it was identified to be downregulated in the parahippocampal area in AD brains. *RNU6–33P* is a non-protein coding gene and it is a pseudogene although it was found to be dysregulated in our analysis. The gene *SNAP25* encodes synaptosome associated protein 25 which is involved in the regulation of neurotransmitter release cycle from synapses, a process which is known to be altered in AD subjects [54]. *SNAP25* was also found in previous research studies to be associated with AD [55]. The gene *VSTM2A* encodes V-set and transmembrane domain containing 2A which was previously reported to be a tumor suppressor although not in brain tissues and some studies found that tumor suppressor responses can be associated with AD [56,57]. The gene *CHN1* encodes Chimerin1, a GTPase-activating protein for ras-related p21-rac and a phorbol ester receptor. It is mainly expressed in neurons and plays an important role in neuronal signal transduction mechanisms and axonal guidance. *CHN1* was never reported as a linked gene to AD, but previous research studies found that disturbances in signal transduction mechanisms are associated with Alzheimer’s disease [58]. The gene *RGS4* encodes regulator of G protein signaling family members which act as GTPase activating proteins. *RGS4* was previously reported as dysregulated in AD [38]. It was also previously reported that there is evidence of dynamic regulations of *RGS4* levels in neuronal systems and many neuropsychiatric disorders are linked to dysfunctions of *RGS* proteins [59]. The mean gene length (in bp) of the top 10 genes is 41760 bp. This mean length agreed with some previous findings on gene length with AD association where they reported that the AD associated genes tend to be large on average [60,61].

The enriched GO annotations in biological processes, molecular functions and cellular components were all related to axonal growth, brain development, the structural constituent of the synapses and neurofilament cytoskeleton organization. Schaffer collateral-CA1 synapse also showed significant enrichment as a cellular component affected in Alzheimer’s disease. As part of the hippocampal structure, it plays a very crucial role in memory formation and information processing. It was previously reported that this synapse is damaged in AD patients [62]. Schaffer collaterals play an important role in the limbic system development that affects learning and memory. Pathway enrichment analysis agreed with the GO annotations where the significantly enriched pathways were mainly related to axon guidance and neuronal system. These pathways were associated with axonal growth and transport. It also showed that the voltage gated potassium channels were affected which signified dysfunctional action potential transmission across the axon. Transmission across chemical synapses and neurotransmitters release cycle were also shown to be affected in AD. All these pathways suggested that axon transmission and synaptic connectivity with other neuronal cells are severely affected in AD. The loss of connectivity between dysfunctional neurons will eventually spread in brain tissues and the affected brain regions will shrink and cause atrophy in the final stages of AD. The downregulated pathways were related to DNA synthesis and elongation. Translation pathway was also downregulated in addition to lipid and protein metabolism pathways which were previously reported in some research studies [63]. The top genes showed a good discriminative power to distinguish between AD and control samples. It was shown using supervised machine learning algorithms that a random forest classifier gave a very good accuracy on the test data. There are many definitions of a “biomarker” which can be broad, controversial and overlapping at the same time [64]. The detected DEGs, especially the top genes, may not be considered as genetic biomarkers for AD since the analysis was mainly limited to late-onset AD subjects. However, they can be considered as biomarkers for axonal transport and synaptic transmission in AD.

The main conclusions from both (HIP+TCx) and (HIP+TCx+PCx) analyses were implicit within the main analysis of all four brain regions. Integrating the gene expression data from four different brain regions gave a more comprehensive results and enabled a broader survey of AD-related gene expression changes, without the constraint of a single brain region. Comparing the three analyses, adding FWM brain region samples increased the number of differentially expressed genes as well as the number of upregulated genes when looking at the brain changes. It increased the statistical power by increasing the number of samples using all 4 brain regions. It was also shown in previous research that some transcripts may increase after the onset of AD symptoms. It is crucial to mention that previous research studies have shown that transcriptional changes for some genes may precede any neuropathy and transcriptional changes can follow up-down or down-up changes [65]. This means that some genes can be downregulated or upregulated before or after any AD symptoms or neuropathy.

One limitation on the classification results is the imbalanced class learning where there were fewer AD samples compared to control samples. This class imbalance may force the classifier to learn most of the target concepts from the majority class with poor learning from the minority class. Synthetic oversampling techniques like SMOTE were not used in order to avoid the possibility of overfitting the training data and to limit the analysis to true biological gene expression data [66]. Undersampling the majority class was also not used in order not to lose information from available data. Otherwise, the accuracy results will be better. The small sample size can also be considered a limitation although LMM deals well with small sample sizes. Another limitation of the study is the inability to study different stages of AD. Advanced staging of the subjects was not available other than the Braak stages. If stratification of the subjects according to their Braak stages was performed, the analysis will be very limited by the sample size especially for AD samples since there were only 15 AD subjects in total and it will not serve or add to the study main purpose of finding AD associated genes. As mentioned before, concerted transcriptional changes can occur before any AD neuropathy, accumulation of amyloid beta and before the onset of AD symptoms [65]. This means that gene expression changes in AD subjects can be detected in very early Braak stages before the onset of AD symptoms [65]. Studying the progression of AD in different brain regions is also a study limitation. It is beyond the scope and capability of this analysis to address the question in which brain region the changes occur first given the fact that the data is not longitudinal and without advanced staging.

## Conclusion

Gene expression analysis with linear mixed models integrating multiple brain regions found multiple AD-associated differentially expressed genes. These genes are mainly related to axon transport and synaptic transmission which affect the neuronal connectivity between cognitive systems involved in AD pathogenesis. These dysregulated genes can help in understanding the etiologies underlying AD progression and open new ways to further explore AD treatment and early diagnosis.

## Supplementary Material

Supplementary Material

## Figures and Tables

**Table 1:** Summary of demographics and characteristics for subjects with and without AD.

Category	Control Subjects	Alzheimer’s Subjects
	Mean (SD)	Mean (SD)
Age at death (yrs)	87.13 (5.62)	89.60 (6.21)
Education (yrs)	14.70 (3.13)	13.67 (2.76)
Braak stage	2.67 (1.24)	4.13 (1.84)
CERAD score	1.16 (0.94)	1.80 (1.32)
	**Count**	**Count**
Sex	14 F/16 M	6 F/9 M
*APOE* Ɛ4 alleles	3 Yes/ 27 No (10 %)	5 Yes/10 No (33.33%)

**Table 2:** Top 10 differentially expressed genes between disease and control samples.

Entrez ID	Symbol	LogFC	AvExp	t	P-value	B
4744	*NEFH*	0.653	4.387	14.34	5.12E-42	94.387
5816	*PVALB*	0.631	2.839	9.60	7.80E-39	86.539
9840	*TESPA1*	0.587	3.437	12.91	6.00E-34	75.185
1.01E+08	*RNU6-33P*	0.574	1.014	12.62	1.86E-32	71.555
6616	*SNAP25*	0.560	8.954	12.3	8.17E-31	67.650
222008	*VSTM2A*	0.551	4.364	12.11	6.87E-30	65.397
9118	*INA*	0.544	4.665	11.95	3.89E-29	63.556
1123	*CHN1*	0.529	7.890	8.43	7.51E-27	58.283
4747	*NEFL*	0.538	6.488	8.42	5.15E-26	56.246
5999	*RGS4*	0.512	5.509	11.26	9.18E-25	55.646
**Symbol**	**Gene Name**		**Gene Length (BP)**		**Chromosome**	
NEFH	Neurofilament, heavy polypeptide		11160		22	
PVALB	Parvalbumin		18795		22	
TESPA1	Thymocyte expressed, positive selection associated		136747		12	
RNU6-33P	RNA, U6 small nuclear 33, pseudogene		106		4	
SNAP25	Synaptosomal-associated protein, 25kDa		88588		20	
VSTM2A	V-set and transmembrane domain containing 2A		28755		7	
INA	Internexin neuronal intermediate filament protein, alpha		13208		10	
CHN1	Chimerin		1206068		2	
NEFL	Neurofilament, light polypeptide		6155		8	
*RGS4*	Regulator of G-protein Signaling		48027		1	

**Table 3:** GO annotations using the top genes and the enriched pathways.

Biological Process GO Terms	P-value	
Postsynaptic intermediate filament cytoskeleton organization	9.56E-06	
Neurofilament cytoskeleton organization	5.73E-05	
Postsynaptic cytoskeleton organization	2.17E-04	
Intermediate filament cytoskeleton organization	8.73E-03	
Intermediate filament-based process	9.28E-03	
Neurofilament bundle assembly	1.43E-02	
**Molecular Function GO Terms**	**P-value**	
Structural constituent of postsynaptic intermediate filament cytoskeleton	3.00E-06	
Structural constituent of synapse	1.99E-04	
Structural constituent of cytoskeleton	2.52E-02	
**Cellular Component GO Terms**	**P-value**	
Neurofilament	2.82E-05	
Postsynaptic intermediate filament cytoskeleton	1.40E-03	
Schaffer collateral – CA1 synapse	8.07E-03	
Postsynaptic cytoskeleton	1.05E-02	
**Upregulated pathways in phenotype AD**	**Gene Set Size**	**Enrichment Score**
Neuronal system	276	2.66
Transmission across chemical synapses	184	2.5
Voltage gated potassium channels	43	2.44
Potassium channels	98	2.43
Neurotransmitter receptor binding and transmission in postsynaptic cell	135	2.34
Neurotransmitter release cycle	34	2.11
Axon guidance	243	1.7
**Down regulated pathways in phenotype AD**	**Gene Set Size**	**Enrichment Score**
Translation	209	−2.6
SRP dependent cotranslational protein targeting to membrane	169	−2.58
Peptide chain elongation	145	−2.5
Metabolism of RNA	316	−2.26
DNA strand elongation	30	−1.93
Synthesis of DNA	91	−1.91
Metabolism of proteins	484	−1.9
Lipoprotein metabolism	28	−1.88
