# Supplementary material for: Identification of Dysregulated Genes for Late-Onset Alzheimer’s Disease Using Gene Expression Data in Brain
Source: J Alzheimers Dis Parkinsonism. Author manuscript; Available in PMC 2021 Jan 1. (PMC7717689)
Supplement: Supplementary Material [file NIHMS1641561-supplement-Supplementary_Material.pdf]

Supplemental Figure and Tables

Supplemental Figure 1: A zoomed-out network graph showing the enriched gene sets where the red nodes represent the up regulated pathways in AD and blue nodes represent the down regulated pathways in AD (up regulated in Control) with a P-value<.01 and FDR<.02.

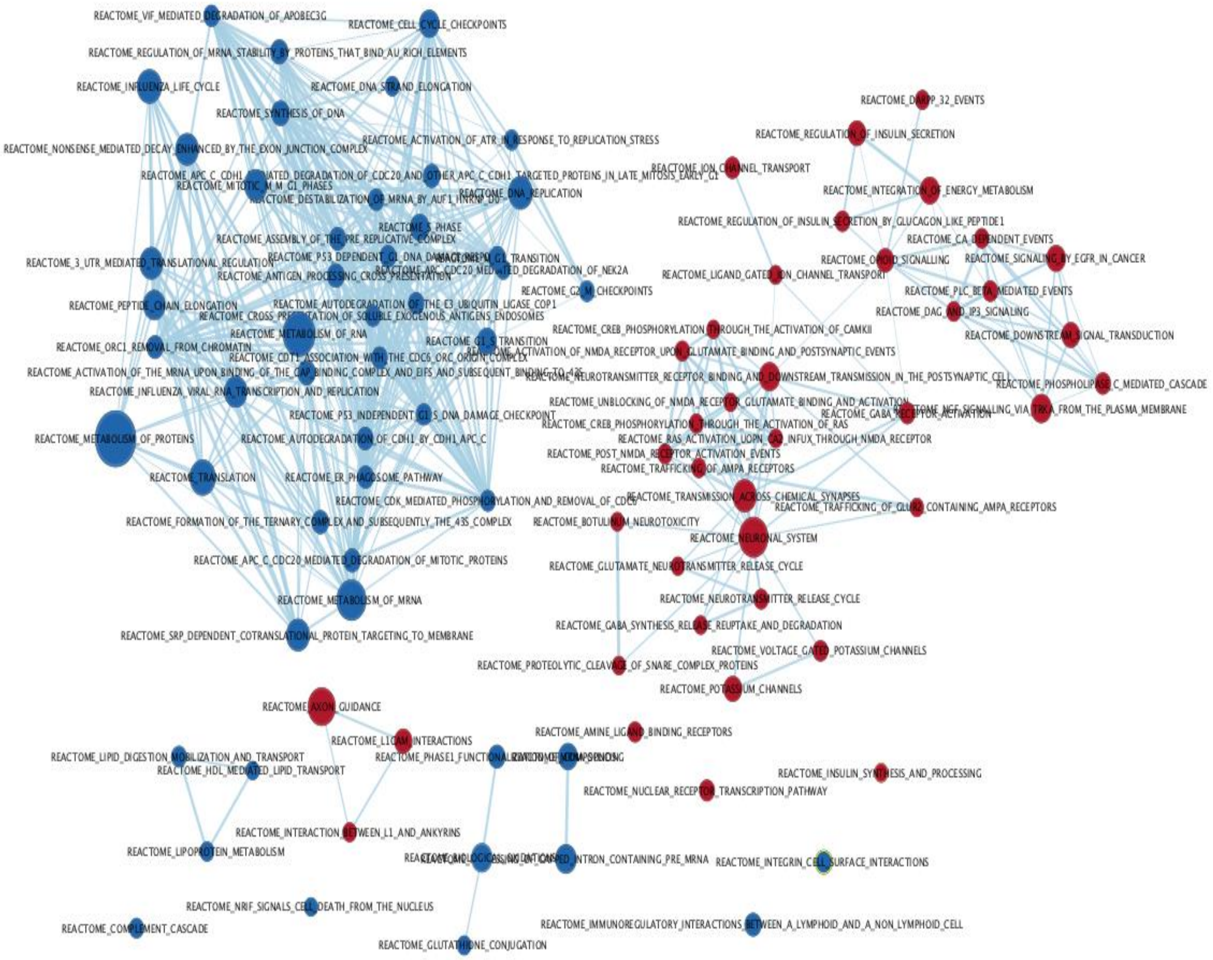

Supplemental Table 1: The 602 differentially expressed genes using the four brain regions sorted using P-values.

Gene Symbol	Status in AD	Sorted Corrected P-Value	Category
NEFH	UpRegulated	5.13E-42	Protein Coding
PVALB	UpRegulated	7.80E-39	Protein Coding
TESPA1	UpRegulated	6.00E-34	Protein Coding
RNU6-33P	UpRegulated	1.86E-32	PseudoGene
SNAP25	UpRegulated	8.17E-31	Protein Coding

VSTM2A	UpRegulated	6.87E-30	Protein Coding
INA	UpRegulated	3.89E-29	Protein Coding
CHN1	UpRegulated	7.51E-27	Protein Coding
NEFL	UpRegulated	5.15E-26	Protein Coding
RGS4	UpRegulated	9.18E-25	Protein Coding
VSNL1	UpRegulated	1.28E-24	Protein Coding
SYT2	UpRegulated	1.15E-23	Protein Coding
NEFM	UpRegulated	1.22E-23	Protein Coding
RPS23P8	DownRegulated	2.15E-23	PseudoGene
KCNS1	UpRegulated	3.45E-23	Protein Coding
CHGA	UpRegulated	4.20E-23	Protein Coding
ENC1	UpRegulated	3.95E-22	Protein Coding
KCNC2	UpRegulated	8.52E-22	Protein Coding
STMN2	UpRegulated	7.78E-21	Protein Coding
GABRA1	UpRegulated	2.33E-20	Protein Coding
TP53INP2	DownRegulated	1.45E-18	Protein Coding
SCN1A	UpRegulated	1.46E-18	Protein Coding
KCNC1	UpRegulated	3.63E-18	Protein Coding
HBB	DownRegulated	4.49E-18	Protein Coding
GABRD	UpRegulated	3.08E-17	Protein Coding
GABRG2	UpRegulated	3.18E-17	Protein Coding
SH3GL2	UpRegulated	4.43E-17	Protein Coding
CCK	UpRegulated	9.23E-17	Protein Coding
SNORD116-16	UpRegulated	2.53E-16	RNA Gene
SNORD89	UpRegulated	2.53E-16	RNA Gene
KCNQ5	UpRegulated	2.95E-16	Protein Coding
HAPLN4	UpRegulated	3.41E-16	Protein Coding
TIAM2	UpRegulated	3.87E-16	Protein Coding
SYT1	UpRegulated	8.38E-16	Protein Coding
NRGN	UpRegulated	1.01E-15	Protein Coding
CKMT1A	UpRegulated	1.23E-15	Protein Coding
KCNA2	UpRegulated	2.39E-15	Protein Coding
THY1	UpRegulated	2.85E-15	Protein Coding
LOC646890	DownRegulated	3.70E-15	NO INFO
GABRB2	UpRegulated	4.53E-15	Protein Coding
SNAP91	UpRegulated	7.07E-15	Protein Coding
MAP2	UpRegulated	9.72E-15	Protein Coding
OLFM1	UpRegulated	1.55E-14	Protein Coding
SYT13	UpRegulated	1.88E-14	Protein Coding
TSPAN13	UpRegulated	2.42E-14	Protein Coding
ELAVL4	UpRegulated	3.18E-14	Protein Coding
CUX2	UpRegulated	3.39E-14	Protein Coding

EPHB6	UpRegulated	7.01E-14	Protein Coding
RAB3C	UpRegulated	1.38E-13	Protein Coding
SYN2	UpRegulated	1.78E-13	Protein Coding
TBR1	UpRegulated	1.95E-13	Protein Coding
NGB	UpRegulated	3.00E-13	Protein Coding
CABP1	UpRegulated	5.20E-13	Protein Coding
RIMS3	UpRegulated	8.98E-13	Protein Coding
GABBR2	UpRegulated	1.13E-12	Protein Coding
SNORA14B	UpRegulated	1.52E-12	RNA Gene
KIF5A	UpRegulated	1.79E-12	Protein Coding
SYT4	UpRegulated	1.89E-12	Protein Coding
LOC102723728	DownRegulated	1.97E-12	Protein Coding
MAST1	UpRegulated	2.00E-12	Protein Coding
MOBP	DownRegulated	2.24E-12	Protein Coding
LOC400927-CSNK1E	DownRegulated	2.38E-12	Protein Coding
OPALIN	DownRegulated	3.14E-12	Protein Coding
AMPH	UpRegulated	4.00E-12	Protein Coding
RGS7BP	UpRegulated	4.20E-12	Protein Coding
TRNV	UpRegulated	5.42E-12	Protein Coding
ELAVL2	UpRegulated	6.30E-12	Protein Coding
MEF2C	UpRegulated	6.48E-12	Protein Coding
MAL	DownRegulated	8.12E-12	Protein Coding
SPTSSB	UpRegulated	1.01E-11	Protein Coding
KIAA1549L	UpRegulated	1.18E-11	Protein Coding
ND5	DownRegulated	1.27E-11	Protein Coding
LINC01123	UpRegulated	1.57E-11	RNA Gene
DNM1	UpRegulated	2.82E-11	Protein Coding
GLRB	UpRegulated	2.99E-11	Protein Coding
SLC12A5	UpRegulated	3.58E-11	Protein Coding
EPHA4	UpRegulated	5.15E-11	Protein Coding
FGF12	UpRegulated	6.26E-11	Protein Coding
IPCEF1	UpRegulated	7.02E-11	Protein Coding
ATP2B2	UpRegulated	7.90E-11	Protein Coding
ATP1A3	UpRegulated	1.01E-10	Protein Coding
TMEM155	UpRegulated	1.19E-10	Protein Coding
SULT4A1	UpRegulated	1.31E-10	Protein Coding
PANX2	UpRegulated	1.50E-10	Protein Coding
ST8SIA3	UpRegulated	2.35E-10	Protein Coding
CPLX2	UpRegulated	2.40E-10	Protein Coding
FAM19A2	UpRegulated	2.41E-10	Protein Coding
RPH3A	UpRegulated	2.56E-10	Protein Coding
TRNQ	UpRegulated	2.58E-10	RNA Gene

CAMK2A	UpRegulated	2.73E-10	Protein Coding
MLLT11	UpRegulated	2.79E-10	Protein Coding
SLC17A6	UpRegulated	3.19E-10	Protein Coding
KCNA1	UpRegulated	3.63E-10	Protein Coding
FLRT2	UpRegulated	4.52E-10	Protein Coding
PDP1	UpRegulated	4.55E-10	Protein Coding
KCNJ6	UpRegulated	4.71E-10	Protein Coding
COPG2IT1	UpRegulated	4.91E-10	RNA Gene
PRSS3	UpRegulated	7.67E-10	Protein Coding
C1orf115	UpRegulated	7.96E-10	Protein Coding
MGAT5B	UpRegulated	8.39E-10	Protein Coding
PTPRT	UpRegulated	9.06E-10	Protein Coding
B4GALNT1	UpRegulated	1.35E-09	Protein Coding
ADAMTS18	DownRegulated	1.51E-09	Protein Coding
CPNE9	UpRegulated	2.07E-09	Protein Coding
KCNV1	UpRegulated	2.38E-09	Protein Coding
KCTD16	UpRegulated	2.47E-09	Protein Coding
PLXNA4	UpRegulated	2.75E-09	Protein Coding
GAP43	UpRegulated	2.96E-09	Protein Coding
RGPD6	UpRegulated	3.33E-09	Protein Coding
RBFOX1	UpRegulated	4.30E-09	Protein Coding
TTR	DownRegulated	4.75E-09	Protein Coding
KCNH8	DownRegulated	5.11E-09	Protein Coding
ITPKA	UpRegulated	5.82E-09	Protein Coding
RBFOX3	UpRegulated	6.99E-09	Protein Coding
RGS10	DownRegulated	8.45E-09	Protein Coding
NAPB	UpRegulated	9.12E-09	Protein Coding
CCL3	UpRegulated	1.14E-08	Protein Coding
LOC105379203	UpRegulated	1.18E-08	RNA Gene
BSN	UpRegulated	1.22E-08	Protein Coding
PCP4	UpRegulated	1.30E-08	Protein Coding
LOC105377390	UpRegulated	1.32E-08	RNA Gene
SMYD2	UpRegulated	1.39E-08	Protein Coding
PPFIA4	UpRegulated	1.59E-08	Protein Coding
HLF	UpRegulated	1.69E-08	Protein Coding
GALNT15	DownRegulated	1.81E-08	Protein Coding
ZMAT4	UpRegulated	2.51E-08	Protein Coding
KCNH5	UpRegulated	2.56E-08	Protein Coding
LNX1	UpRegulated	2.70E-08	Protein Coding
NRSN1	UpRegulated	2.79E-08	Protein Coding
EEF1A2	UpRegulated	2.89E-08	Protein Coding
SLC4A10	UpRegulated	2.91E-08	Protein Coding

CTNNA3	DownRegulated	2.97E-08	Protein Coding
GABRA4	UpRegulated	3.30E-08	Protein Coding
C11orf87	UpRegulated	3.45E-08	Protein Coding
TNFRSF25	UpRegulated	3.54E-08	Protein Coding
TRHDE	UpRegulated	4.16E-08	Protein Coding
SYN1	UpRegulated	5.73E-08	Protein Coding
PLLP	DownRegulated	6.12E-08	Protein Coding
MRAP2	UpRegulated	7.76E-08	Protein Coding
KCNT1	UpRegulated	8.10E-08	Protein Coding
HSD17B3	DownRegulated	8.55E-08	Protein Coding
FIBCD1	DownRegulated	8.80E-08	Protein Coding
KCNH7	UpRegulated	9.18E-08	Protein Coding
FAM57B	UpRegulated	9.58E-08	Protein Coding
RNVU1-10	UpRegulated	1.04E-07	RNA Gene
NCEH1	UpRegulated	1.12E-07	Protein Coding
KCNB1	UpRegulated	1.16E-07	Protein Coding
HBA1	DownRegulated	1.92E-07	Protein Coding
MIR124-2HG	UpRegulated	2.10E-07	RNA Gene
EMX1	UpRegulated	2.22E-07	Protein Coding
L1CAM	UpRegulated	2.27E-07	Protein Coding
CBLN2	UpRegulated	2.61E-07	Protein Coding
DGCR5	UpRegulated	2.69E-07	RNA Gene
SNX10	UpRegulated	2.89E-07	Protein Coding
SCN1B	UpRegulated	2.90E-07	Protein Coding
KIF3C	UpRegulated	3.03E-07	Protein Coding
RPL41P5	DownRegulated	3.05E-07	PseudoGene
ARPP21	UpRegulated	3.20E-07	Protein Coding
FAM84A	UpRegulated	3.36E-07	Protein Coding
BASP1	UpRegulated	3.69E-07	Protein Coding
KCNJ4	UpRegulated	3.74E-07	Protein Coding
HTR2A	UpRegulated	3.81E-07	Protein Coding
KCNIP4-IT1	UpRegulated	6.68E-07	RNA Gene
MAG	DownRegulated	6.77E-07	Protein Coding
RGS6	UpRegulated	6.91E-07	Protein Coding
HECW1	UpRegulated	7.35E-07	Protein Coding
LIX1	DownRegulated	7.73E-07	Protein Coding
CRLF1	UpRegulated	8.85E-07	Protein Coding
FAXC	UpRegulated	9.43E-07	Protein Coding
VSTM2A-OT1	UpRegulated	9.53E-07	RNA Gene
PRKCB	UpRegulated	1.01E-06	Protein Coding
PAQR6	DownRegulated	1.01E-06	Protein Coding
SCN8A	UpRegulated	1.03E-06	Protein Coding

CREG2	UpRegulated	1.12E-06	Protein Coding
SNORD116-15	UpRegulated	1.16E-06	RNA Gene
PIP5K1C	UpRegulated	1.16E-06	Protein Coding
KCNQ5-IT1	UpRegulated	1.23E-06	RNA Gene
SNORA43	UpRegulated	1.43E-06	RNA Gene
STXBP1	UpRegulated	1.52E-06	Protein Coding
SCN4B	UpRegulated	1.53E-06	Protein Coding
FAM163B	UpRegulated	1.96E-06	Protein Coding
LINC00844	DownRegulated	2.08E-06	RNA Gene
CDS1	UpRegulated	2.09E-06	Protein Coding
RTN4R	UpRegulated	2.16E-06	Protein Coding
ZNF697	UpRegulated	2.16E-06	Protein Coding
ESRRG	UpRegulated	2.36E-06	Protein Coding
NGEF	UpRegulated	2.37E-06	Protein Coding
CDH12	UpRegulated	2.57E-06	Protein Coding
LOC100418883	DownRegulated	2.78E-06	PseudoGene
EFNA5	UpRegulated	2.80E-06	Protein Coding
OPN3	UpRegulated	2.80E-06	Protein Coding
CDH22	UpRegulated	2.95E-06	Protein Coding
SOSTDC1	UpRegulated	2.95E-06	Protein Coding
GABRB3	UpRegulated	3.03E-06	Protein Coding
PRDM8	UpRegulated	3.06E-06	Protein Coding
HCN1	UpRegulated	3.12E-06	Protein Coding
SEPTIN4	DownRegulated	3.17E-06	Protein Coding
RORB	UpRegulated	3.19E-06	Protein Coding
GRIN1	UpRegulated	3.44E-06	Protein Coding
VWA7	UpRegulated	3.57E-06	Protein Coding
TMEM151B	UpRegulated	3.79E-06	Protein Coding
CCDC3	UpRegulated	3.83E-06	Protein Coding
SNORD116-3	UpRegulated	3.83E-06	RNA Gene
SNORD116-9	UpRegulated	3.83E-06	RNA Gene
RSPO2	UpRegulated	3.96E-06	Protein Coding
AQP1	DownRegulated	4.24E-06	Protein Coding
LINC00622	UpRegulated	4.26E-06	RNA Gene
FSTL4	UpRegulated	4.50E-06	Protein Coding
MPP7	UpRegulated	4.61E-06	Protein Coding
RNF144A-AS1	UpRegulated	5.05E-06	RNA Gene
KCNIP2	UpRegulated	5.42E-06	Protein Coding
SYT7	UpRegulated	5.45E-06	Protein Coding
EPHX4	UpRegulated	5.88E-06	Protein Coding
PABPC1P11	UpRegulated	5.89E-06	PseudoGene
CIT	UpRegulated	6.15E-06	Protein Coding

SPARC	DownRegulated	6.32E-06	Protein Coding
SYNPO	UpRegulated	6.38E-06	Protein Coding
ZFP57	DownRegulated	6.56E-06	Protein Coding
ALDH1A1	DownRegulated	8.09E-06	Protein Coding
LINC00599	UpRegulated	8.27E-06	RNA Gene
NPTX1	UpRegulated	8.62E-06	Protein Coding
PRKAR2B	UpRegulated	9.08E-06	Protein Coding
NAP1L2	UpRegulated	9.41E-06	Protein Coding
ABHD8	UpRegulated	9.72E-06	Protein Coding
APLNR	DownRegulated	9.75E-06	Protein Coding
NEUROD2	UpRegulated	9.95E-06	Protein Coding
SV2B	UpRegulated	1.11E-05	Protein Coding
SNORD3D	UpRegulated	1.17E-05	RNA Gene
MAP7D2	UpRegulated	1.22E-05	Protein Coding
VAV3	UpRegulated	1.30E-05	Protein Coding
SMOC1	DownRegulated	1.35E-05	Protein Coding
SLC17A7	UpRegulated	1.39E-05	Protein Coding
COL4A5	DownRegulated	1.81E-05	Protein Coding
SNORD116-8	UpRegulated	2.04E-05	RNA Gene
ROS1	UpRegulated	2.11E-05	Protein Coding
FOLH1	DownRegulated	2.23E-05	Protein Coding
KNDC1	UpRegulated	2.28E-05	Protein Coding
ISLR	UpRegulated	2.29E-05	Protein Coding
TMEM35	UpRegulated	2.35E-05	Protein Coding
LRRC4	UpRegulated	2.36E-05	Protein Coding
LPCAT4	UpRegulated	2.42E-05	Protein Coding
CITED2	UpRegulated	2.42E-05	Protein Coding
RXFP1	UpRegulated	2.45E-05	Protein Coding
CACNB4	UpRegulated	2.51E-05	Protein Coding
LOC105379480	UpRegulated	2.52E-05	NO INFO
LOC105372217	DownRegulated	2.59E-05	RNA Gene
LOC105373721	UpRegulated	2.60E-05	NO INFO
OLFM3	UpRegulated	2.67E-05	Protein Coding
LAMP5	UpRegulated	2.88E-05	Protein Coding
CPLX1	UpRegulated	2.92E-05	Protein Coding
TXNIP	DownRegulated	3.10E-05	Protein Coding
RARB	UpRegulated	3.31E-05	Protein Coding
B4GALT6	UpRegulated	3.48E-05	Protein Coding
JPH3	UpRegulated	3.51E-05	Protein Coding
PAIP2B	DownRegulated	3.60E-05	Protein Coding
CADPS	UpRegulated	3.65E-05	Protein Coding
LOC105369862	UpRegulated	3.73E-05	NO INFO

TMEM144	DownRegulated	3.80E-05	Protein Coding
LRP3	UpRegulated	3.85E-05	Protein Coding
YWHAH	UpRegulated	4.08E-05	Protein Coding
LOC105376654	UpRegulated	4.21E-05	RNA Gene
DLGAP1-AS4	UpRegulated	4.40E-05	RNA Gene
FABP5P7	DownRegulated	4.56E-05	PseudoGene
SLC14A1	DownRegulated	4.63E-05	Protein Coding
DRD1	UpRegulated	4.73E-05	Protein Coding
IMPDH1P11	UpRegulated	5.02E-05	PseudoGene
TAGLN3	UpRegulated	5.02E-05	Protein Coding
CERCAM	DownRegulated	5.07E-05	Protein Coding
SNORD116-22	UpRegulated	5.37E-05	RNA Gene
SNCB	UpRegulated	5.58E-05	Protein Coding
DLGAP1	UpRegulated	5.81E-05	Protein Coding
OR2A9P	DownRegulated	5.90E-05	PseudoGene
ADAM23	UpRegulated	5.94E-05	Protein Coding
CELF4	UpRegulated	6.08E-05	Protein Coding
CACNB3	UpRegulated	6.17E-05	Protein Coding
SLC26A4-AS1	UpRegulated	6.54E-05	RNA Gene
NCDN	UpRegulated	6.59E-05	Protein Coding
CLVS2	UpRegulated	6.94E-05	Protein Coding
PITPNM1	UpRegulated	6.94E-05	Protein Coding
ARRDC4	DownRegulated	7.59E-05	Protein Coding
SNORD116-20	UpRegulated	7.84E-05	RNA Gene
CNP	DownRegulated	8.48E-05	Protein Coding
MOG	DownRegulated	8.61E-05	Protein Coding
LYPD8	UpRegulated	8.62E-05	Protein Coding
NELL2	UpRegulated	8.65E-05	Protein Coding
TUBA4A	UpRegulated	9.19E-05	Protein Coding
STK19B	DownRegulated	9.20E-05	PseudoGene
PHLDB1	DownRegulated	9.83E-05	Protein Coding
SCRT1	UpRegulated	9.93E-05	Protein Coding
CLSTN2	UpRegulated	9.95E-05	Protein Coding
FLRT3	UpRegulated	9.96E-05	Protein Coding
CDK5R1	UpRegulated	0.000102266	Protein Coding
THRB	UpRegulated	0.000103762	Protein Coding
ABLIM2	UpRegulated	0.000104943	Protein Coding
LOC645166	UpRegulated	0.00011081	Protein Coding
CSF1R	DownRegulated	0.000112546	Protein Coding
CACNG2	UpRegulated	0.000113274	Protein Coding
SPTBN4	UpRegulated	0.000116165	Protein Coding
LINC01106	UpRegulated	0.000122079	RNA Gene

ANK1	UpRegulated	0.000122567	Protein Coding
DEAR	UpRegulated	0.000128043	RNA Gene
RIMKLA	UpRegulated	0.000133446	Protein Coding
LOC101929800	DownRegulated	0.000139017	RNA Gene
AMPD3	DownRegulated	0.000140647	Protein Coding
ELOVL4	UpRegulated	0.00014212	Protein Coding
LOC101929617	UpRegulated	0.000146421	RNA Gene
LUZP1	UpRegulated	0.000147068	Protein Coding
RNA5S9	UpRegulated	0.000149168	RNA Gene
UBE2QL1	UpRegulated	0.000152192	Protein Coding
PTER	UpRegulated	0.000170282	Protein Coding
ZNF385B	UpRegulated	0.000170983	Protein Coding
MIR4440	UpRegulated	0.000180068	RNA Gene
ADARB1	UpRegulated	0.000183877	Protein Coding
ADGRL2	UpRegulated	0.000189351	Protein Coding
CAHM	DownRegulated	0.000190132	RNA Gene
SYBU	UpRegulated	0.000208664	Protein Coding
JPH4	UpRegulated	0.000213415	Protein Coding
SNPH	UpRegulated	0.000238841	Protein Coding
MAL2	UpRegulated	0.000244347	Protein Coding
MTCO1P2	DownRegulated	0.000247028	PseudoGene
LOC105369302	UpRegulated	0.000264598	RNA Gene
ANGPTL4	DownRegulated	0.000272382	Protein Coding
ADD2	UpRegulated	0.000276333	Protein Coding
CTXN2	UpRegulated	0.000294773	Protein Coding
RASD2	UpRegulated	0.000306383	Protein Coding
STEAP2	UpRegulated	0.000314697	Protein Coding
SCN2A	UpRegulated	0.000319435	Protein Coding
CD9	DownRegulated	0.000327382	Protein Coding
NSG1	UpRegulated	0.000334975	Protein Coding
KRT222	UpRegulated	0.000345584	Protein Coding
GRM1	UpRegulated	0.00034833	Protein Coding
LOC105372855	UpRegulated	0.000349764	RNA Gene
CCDC64	UpRegulated	0.000350812	Protein Coding
EPDR1	UpRegulated	0.0003582	Protein Coding
ADRB1	UpRegulated	0.000363221	Protein Coding
RALYL	UpRegulated	0.000393146	Protein Coding
CLSTN3	UpRegulated	0.000394291	Protein Coding
LDB2	UpRegulated	0.000398858	Protein Coding
LRFN5	UpRegulated	0.000415477	Protein Coding
NPIPA8	DownRegulated	0.000442618	Protein Coding
ARHGAP44	UpRegulated	0.00049941	Protein Coding

RNF208	UpRegulated	0.000500354	Protein Coding
SHROOM2	UpRegulated	0.000526712	Protein Coding
LOC102723645	UpRegulated	0.000530655	RNA Gene
PPP3CA	UpRegulated	0.000536257	Protein Coding
EFHD2	UpRegulated	0.000536719	Protein Coding
PRKAR1B	UpRegulated	0.000541598	Protein Coding
MYT1L	UpRegulated	0.000562487	Protein Coding
SEPTIN3	UpRegulated	0.000584428	Protein Coding
HSPA1B	DownRegulated	0.000589925	Protein Coding
STX1B	UpRegulated	0.000594503	Protein Coding
LOC105373027	DownRegulated	0.000594564	RNA Gene
MBP	DownRegulated	0.000599966	Protein Coding
KCNH1	UpRegulated	0.000625078	Protein Coding
MAPRE1P1	UpRegulated	0.000631433	PseudoGene
PLEKHH1	DownRegulated	0.000633208	Protein Coding
LOC105376687	UpRegulated	0.000690691	RNA Gene
MICAL2	UpRegulated	0.000709722	Protein Coding
ELK1	UpRegulated	0.000722166	Protein Coding
HSD3BP4	UpRegulated	0.000725967	PseudoGene
NCALD	UpRegulated	0.000768851	Protein Coding
PI16	DownRegulated	0.000769447	Protein Coding
GALNT9	UpRegulated	0.000777715	Protein Coding
SYNGR3	UpRegulated	0.000791232	Protein Coding
VSTM2L	UpRegulated	0.000806079	Protein Coding
LOC101928832	DownRegulated	0.000815654	RNA Gene
DMTN	UpRegulated	0.000824358	Protein Coding
KCNK12	UpRegulated	0.000867401	Protein Coding
NPTN	UpRegulated	0.000875164	Protein Coding
KCNQ2	UpRegulated	0.000893507	Protein Coding
TRNP1	UpRegulated	0.000930425	Protein Coding
PLEKHG5	UpRegulated	0.000959668	Protein Coding
MIAT	UpRegulated	0.000965972	RNA Gene
ADAM28	DownRegulated	0.000979707	Protein Coding
UNC5A	UpRegulated	0.001015155	Protein Coding
LOC101927119	DownRegulated	0.00105621	NO INFO
LOC102724457	UpRegulated	0.001068605	NO INFO
FGF17	UpRegulated	0.001075686	Protein Coding
SATB1	UpRegulated	0.001133815	Protein Coding
CKMT1B	UpRegulated	0.001135783	Protein Coding
LOC102724238	DownRegulated	0.001171418	RNA Gene
ARHGAP32	UpRegulated	0.001179855	Protein Coding
PDE1B	UpRegulated	0.001191167	Protein Coding

DIRAS1	UpRegulated	0.001222845	Protein Coding
CNST	UpRegulated	0.001234773	Protein Coding
CBLN4	UpRegulated	0.001237615	Protein Coding
HMP19	UpRegulated	0.00129365	Protein Coding
SV2C	UpRegulated	0.00133713	Protein Coding
CARNS1	DownRegulated	0.001366849	Protein Coding
PCDH7	UpRegulated	0.001381717	Protein Coding
PHYHIP	UpRegulated	0.001385825	Protein Coding
CABP7	DownRegulated	0.001403199	Protein Coding
FAM171A2	UpRegulated	0.001424906	Protein Coding
LGR5	DownRegulated	0.001426869	Protein Coding
ANKRD20A19P	DownRegulated	0.001485487	PseudoGene
TMEM63A	DownRegulated	0.001594322	Protein Coding
ITPR1	UpRegulated	0.001637622	Protein Coding
HTR1E	UpRegulated	0.001667235	Protein Coding
PTPN5	UpRegulated	0.001682993	Protein Coding
KCNA3	UpRegulated	0.001721132	Protein Coding
LINCR-0003	UpRegulated	0.001735696	RNA Gene
PCLO	UpRegulated	0.0017896	Protein Coding
CP	DownRegulated	0.001811348	Protein Coding
FBXO41	UpRegulated	0.001826729	Protein Coding
KLHL29	UpRegulated	0.001863138	Protein Coding
VN1R83P	DownRegulated	0.001902185	PseudoGene
KIF19	DownRegulated	0.001907838	Protein Coding
RAB15	UpRegulated	0.001941429	Protein Coding
RTN2	UpRegulated	0.001947116	Protein Coding
OPCML	UpRegulated	0.002065132	Protein Coding
NPM2	UpRegulated	0.002088887	Protein Coding
TTC9B	UpRegulated	0.002107479	Protein Coding
LOC105373224	UpRegulated	0.002126677	RNA Gene
KCNJ3	UpRegulated	0.002176544	Protein Coding
AKAP12	UpRegulated	0.002286946	Protein Coding
LOC101928622	UpRegulated	0.002322633	RNA Gene
ZBTB45P2	UpRegulated	0.002337751	PseudoGene
FGF9	UpRegulated	0.002344803	Protein Coding
NEUROD6	UpRegulated	0.002349917	Protein Coding
ANO3	UpRegulated	0.00246829	Protein Coding
NSF	UpRegulated	0.002529053	Protein Coding
RUSC1	UpRegulated	0.002760119	Protein Coding
ITGB4	DownRegulated	0.00283301	Protein Coding
KCNS2	UpRegulated	0.002843235	Protein Coding
LOC105374516	UpRegulated	0.002860465	RNA Gene

CDH18	UpRegulated	0.002938072	Protein Coding
CEND1	UpRegulated	0.002957455	Protein Coding
ACTL6B	UpRegulated	0.002968864	Protein Coding
CCDC184	UpRegulated	0.003127343	Protein Coding
ABCA8	DownRegulated	0.003129697	Protein Coding
FRMPD4	UpRegulated	0.003150376	RNA Gene
BEST1	DownRegulated	0.003159495	Protein Coding
LOC101929579	DownRegulated	0.003164042	RNA Gene
CACNA1I	UpRegulated	0.003272406	Protein Coding
CMTM5	DownRegulated	0.003292644	Protein Coding
PCSK2	UpRegulated	0.003377388	Protein Coding
AMER3	UpRegulated	0.003436694	Protein Coding
STAC2	UpRegulated	0.003531464	Protein Coding
SNORA45A	UpRegulated	0.003548343	RNA Gene
C1QTNF9B	DownRegulated	0.003565715	Protein Coding
PDK4	DownRegulated	0.003577261	Protein Coding
LRRN3	UpRegulated	0.003730112	Protein Coding
MATK	UpRegulated	0.003866756	Protein Coding
RASL12	DownRegulated	0.003967656	Protein Coding
RPL18AP10	UpRegulated	0.004049925	PseudoGene
GPR158	UpRegulated	0.004161295	RNA Gene
SNHG5	DownRegulated	0.004438642	RNA Gene
PARM1	UpRegulated	0.004501496	RNA Gene
CADM3	UpRegulated	0.004627472	Protein Coding
CHD5	UpRegulated	0.004655926	Protein Coding
TRNP	DownRegulated	0.004774715	Protein Coding
PRR18	DownRegulated	0.004800546	Protein Coding
HEPACAM	DownRegulated	0.004822176	Protein Coding
LOC100996635	UpRegulated	0.004857116	RNA Gene
RPL10P16	DownRegulated	0.00502145	PseudoGene
LOC440896	DownRegulated	0.005369458	PseudoGene
LOC105374518	UpRegulated	0.005539998	NO INFO
CNR1	UpRegulated	0.005555859	Protein Coding
USH1C	DownRegulated	0.005974723	Protein Coding
ERBB3	DownRegulated	0.005998433	Protein Coding
LOC105376710	DownRegulated	0.006203575	RNA Gene
NAIP	DownRegulated	0.006219921	Protein Coding
C9orf16	UpRegulated	0.006311219	Protein Coding
MKX	UpRegulated	0.006343627	Protein Coding
RET	UpRegulated	0.006450618	Protein Coding
RNU1-21P	DownRegulated	0.006471302	PseudoGene
LOC105370610	UpRegulated	0.006497501	RNA Gene

CPNE4	UpRegulated	0.006558434	Protein Coding
LOC100506725	DownRegulated	0.006699351	RNA Gene
CYCSP8	UpRegulated	0.006705452	PseudoGene
PLCB1	UpRegulated	0.006714488	Protein Coding
CASKIN1	UpRegulated	0.006803341	Protein Coding
OPRD1	UpRegulated	0.006972553	Protein Coding
UNC13A	UpRegulated	0.007000571	Protein Coding
ATP2B3	UpRegulated	0.00702227	Protein Coding
GULP1	UpRegulated	0.007032909	Protein Coding
LMTK2	UpRegulated	0.00707264	Protein Coding
KCNC3	UpRegulated	0.007305895	Protein Coding
LOC105378664	UpRegulated	0.007666547	NO INFO
TMEM261P1	UpRegulated	0.007960657	PseudoGene
LOC105373026	DownRegulated	0.008177673	RNA Gene
DLGAP3	UpRegulated	0.008368863	Protein Coding
GFOD1	UpRegulated	0.008448575	Protein Coding
MAP3K10	UpRegulated	0.008463507	Protein Coding
HS6ST3	UpRegulated	0.008740283	Protein Coding
SYT16	UpRegulated	0.008749225	Protein Coding
LRRTM4	UpRegulated	0.008760925	Protein Coding
PLEKHA6	UpRegulated	0.008824186	Protein Coding
GLS	UpRegulated	0.009269547	Protein Coding
HIST1H4K	UpRegulated	0.009293045	Protein Coding
FBXL16	UpRegulated	0.009392171	Protein Coding
GLRA3	UpRegulated	0.009451964	Protein Coding
SGK2	DownRegulated	0.00962258	Protein Coding
CHST1	UpRegulated	0.009979213	Protein Coding
RASL10B	UpRegulated	0.010357391	Protein Coding
CDH9	UpRegulated	0.010595629	Protein Coding
PAK1	UpRegulated	0.010676537	Protein Coding
NCS1	UpRegulated	0.010742081	Protein Coding
ZDHHC22	UpRegulated	0.010922541	Protein Coding
TGFA	DownRegulated	0.011885679	Protein Coding
MTCL1	UpRegulated	0.011889777	PseudoGene
PWAR1	UpRegulated	0.012070507	RNA Gene
SLC2A13	UpRegulated	0.012145317	PseudoGene
AASS	DownRegulated	0.012540202	Protein Coding
RAB6B	UpRegulated	0.012633783	Protein Coding
FAM69C	DownRegulated	0.012724849	Protein Coding
LPPR1	DownRegulated	0.012735858	Protein Coding
LOC105376054	UpRegulated	0.012880022	NO INFO
TUBA8	UpRegulated	0.012926521	Protein Coding

LOC101928092	UpRegulated	0.013135825	RNA Gene
LOC101927008	UpRegulated	0.013135825	RNA Gene
RUNDC3A	UpRegulated	0.013300896	RNA Gene
SHANK3	UpRegulated	0.01334912	Protein Coding
MT1G	UpRegulated	0.013377942	Protein Coding
GCNT4	UpRegulated	0.013394461	Protein Coding
ST6GALNAC5	UpRegulated	0.013642937	Protein Coding
AMMECR1LP1	UpRegulated	0.013653162	Protein Coding
MTUS2	UpRegulated	0.013653605	Protein Coding
LINC00609	DownRegulated	0.013757799	RNA Gene
LDB3	DownRegulated	0.01405155	Protein Coding
FAM135B	UpRegulated	0.014232119	Protein Coding
ADAMTS4	DownRegulated	0.014292482	Protein Coding
NEK3	DownRegulated	0.014429153	Protein Coding
CHGB	UpRegulated	0.014640954	Protein Coding
PLP1	DownRegulated	0.014821515	Protein Coding
CXCL14	UpRegulated	0.014871209	Protein Coding
TGFB3	DownRegulated	0.015141239	Protein Coding
MYRF	DownRegulated	0.015182413	Protein Coding
GPIHBP1	DownRegulated	0.016274289	Protein Coding
SEPTIN5	UpRegulated	0.016290672	Protein Coding
FAM149A	DownRegulated	0.016323635	Protein Coding
RND1	UpRegulated	0.016498833	Protein Coding
SNCG	UpRegulated	0.017181324	Protein Coding
CHRM1	UpRegulated	0.018079147	Protein Coding
RCAN2	UpRegulated	0.018118471	Protein Coding
FRRS1L	UpRegulated	0.018738716	Protein Coding
RIT2	UpRegulated	0.018847221	Protein Coding
KCNAB2	UpRegulated	0.018898874	Protein Coding
CAP2	UpRegulated	0.019025724	Protein Coding
KIAA1522	UpRegulated	0.019583436	Protein Coding
ACTA2	UpRegulated	0.019611062	Protein Coding
FHL2	UpRegulated	0.019650627	Protein Coding
DBNDD1	UpRegulated	0.019907617	Protein Coding
R3HDM1	UpRegulated	0.019986747	Protein Coding
KIAA1644	UpRegulated	0.020200552	Protein Coding
SEMA6B	UpRegulated	0.020959931	Protein Coding
CAMK2B	UpRegulated	0.021437204	Protein Coding
RNASE1	DownRegulated	0.022124791	Protein Coding
LOC102724394	DownRegulated	0.022197729	RNA Gene
EPN3	UpRegulated	0.022377761	Protein Coding
TNC	DownRegulated	0.022994907	Protein Coding

RPL31P25	UpRegulated	0.023334785	PseudoGene
VWC2L	UpRegulated	0.023820731	Protein Coding
RPS29P22	UpRegulated	0.024160096	PseudoGene
CSF3R	DownRegulated	0.024204362	Protein Coding
SLITRK4	UpRegulated	0.024235399	Protein Coding
SYNPR	UpRegulated	0.024284479	Protein Coding
MPPED1	UpRegulated	0.024469307	Protein Coding
WASH5P	UpRegulated	0.02472553	PseudoGene
LOC105374278	UpRegulated	0.025798705	RNA Gene
LOC105374504	UpRegulated	0.025963454	RNA Gene
APOC1	DownRegulated	0.027639614	Protein Coding
REEP2	UpRegulated	0.027811122	Protein Coding
BCL11A	UpRegulated	0.028085794	Protein Coding
RPL39P29	UpRegulated	0.028156888	PseudoGene
NETO1	UpRegulated	0.028375487	Protein Coding
TRHDE-AS1	UpRegulated	0.029228824	RNA Gene
PCDH8	UpRegulated	0.029514642	Protein Coding
LRFN2	UpRegulated	0.030022169	Protein Coding
LOC101929570	UpRegulated	0.031088969	RNA Gene
ABCC4	DownRegulated	0.031243734	Protein Coding
LOC105376918	UpRegulated	0.031264381	RNA Gene
C5orf64	DownRegulated	0.031276246	Protein Coding
CNKSR2	UpRegulated	0.03213095	Protein Coding
RAB27B	UpRegulated	0.032859223	Protein Coding
C3	DownRegulated	0.033053085	Protein Coding
C1QL3	UpRegulated	0.03332314	Protein Coding
NUDT4	UpRegulated	0.033362263	Protein Coding
HIST1H3A	UpRegulated	0.033788432	Protein Coding
SNORD116-2	UpRegulated	0.034921392	RNA Gene
HMGN2P37	UpRegulated	0.036011859	PseudoGene
SNORD55	UpRegulated	0.036738483	RNA Gene
PPP3R1	UpRegulated	0.036841547	Protein Coding
LIN7A	UpRegulated	0.037895762	Protein Coding
SYT3	UpRegulated	0.038338732	Protein Coding
CADPS2	UpRegulated	0.038989145	Protein Coding
RAB11FIP5	UpRegulated	0.0393783	Protein Coding
S100B	DownRegulated	0.040266065	Protein Coding
EXTL2	UpRegulated	0.040670087	Protein Coding
RFPL1	UpRegulated	0.040835787	Protein Coding
LOC644767	UpRegulated	0.041859045	RNA Gene
TOMM34	UpRegulated	0.042375682	Protein Coding
RAP1GAP2	UpRegulated	0.043477518	Protein Coding

SEMA3B	DownRegulated	0.043574229	Protein Coding
PTPRO	UpRegulated	0.044343743	Protein Coding
PDZRN3	UpRegulated	0.045826034	Protein Coding
SV2A	UpRegulated	0.046349603	Protein Coding
SYP	UpRegulated	0.046793123	Protein Coding
ZNF204P	UpRegulated	0.047574524	PseudoGene
LMO4	UpRegulated	0.049273576	Protein Coding
KIAA1045	UpRegulated	0.049732135	Protein Coding
GOLGA7B	UpRegulated	0.049979477	Protein Coding

Supplemental Table 2: The 146 differentially expressed genes using samples from HIP, TCx, and PCx brain regions sorted using P-values.

Gene Symbol	Corrected Sorted Pvalue	Status in AD
RNU6-33P	2.84E-55	UpRegulated
SNORD116-16	1.81E-18	UpRegulated
CCL3	9.44E-17	UpRegulated
LOC102723728	1.95E-14	DownRegulated
LOC101929800	3.27E-14	DownRegulated
PVALB	3.69E-14	UpRegulated
FIBCD1	5.82E-13	DownRegulated
LOC400927-CSNK1E	6.58E-12	DownRegulated
SYT2	6.97E-12	UpRegulated
TRNP	1.98E-11	DownRegulated
FABP5P7	1.92E-10	DownRegulated
OPALIN	4.17E-10	DownRegulated
HBB	1.04E-09	DownRegulated
MTCO1P2	4.19E-09	DownRegulated
LIX1	8.77E-09	DownRegulated
SNORA14B	1.30E-08	UpRegulated
SLC14A1	2.26E-08	DownRegulated
LOC440896	4.37E-08	DownRegulated
C1QTNF9B	4.47E-08	DownRegulated
ANKRD20A19P	1.95E-07	DownRegulated
TESPA1	2.30E-07	UpRegulated
CD9	2.68E-07	DownRegulated
TMEM144	3.53E-07	DownRegulated
RNVU1-10	4.42E-07	UpRegulated
RPL15P3	6.64E-07	UpRegulated
ALDH1A1	7.51E-07	DownRegulated
SNORA2A	7.67E-07	DownRegulated
IL12RB2	1.01E-06	DownRegulated
RNU1-21P	1.46E-06	DownRegulated

RGS10	2.31E-06	DownRegulated
RPS23P8	2.77E-06	DownRegulated
SNORD116-8	3.34E-06	UpRegulated
OR2A9P	4.06E-06	DownRegulated
LOC105374506	4.60E-06	DownRegulated
NEFH	5.07E-06	UpRegulated
PLP1	5.63E-06	DownRegulated
NGB	5.94E-06	UpRegulated
MAL	6.21E-06	DownRegulated
CABP7	6.83E-06	DownRegulated
RN7SL4P	7.75E-06	UpRegulated
HIST1H4K	8.46E-06	UpRegulated
LOC105379480	1.14E-05	UpRegulated
SNORA24	1.45E-05	DownRegulated
AQP1	1.83E-05	DownRegulated
ACTA2	1.92E-05	UpRegulated
GALNT15	2.09E-05	DownRegulated
RN7SL5P	3.00E-05	UpRegulated
PLLP	3.35E-05	DownRegulated
LOC101929179	3.43E-05	DownRegulated
DDX39B	4.97E-05	DownRegulated
KCNS1	5.10E-05	UpRegulated
LOC105379032	7.34E-05	DownRegulated
FNDC1	7.41E-05	DownRegulated
CD38	0.000103671	DownRegulated
HIST1H2BM	0.000111217	DownRegulated
CP	0.000119102	DownRegulated
LOC646890	0.00011921	DownRegulated
HBA1	0.000140027	DownRegulated
FAM183A	0.000150969	DownRegulated
RPL41P5	0.000248737	DownRegulated
CRABP1	0.000252113	UpRegulated
EIF4EP2	0.000271835	DownRegulated
HMGN2P37	0.000279942	UpRegulated
LPPR1	0.000283393	DownRegulated
CLDN11	0.00028707	DownRegulated
ADIRF	0.000302805	DownRegulated
MOG	0.000349632	DownRegulated
EMP1	0.000467551	DownRegulated
SNORD8	0.000491912	UpRegulated
LYPD1	0.00055676	DownRegulated
FPGT-TNNI3K	0.000601077	DownRegulated

SNORA27	0.000629633	DownRegulated
SNORD89	0.000722489	UpRegulated
S100B	0.000728841	DownRegulated
SERF1A	0.000827663	DownRegulated
LINC00844	0.000894145	DownRegulated
NPIPA8	0.000898784	DownRegulated
TXNIP	0.000930545	DownRegulated
MOBP	0.000932856	DownRegulated
MIR4441	0.000978469	DownRegulated
LOC642423	0.000997141	UpRegulated
TRNV	0.00130296	UpRegulated
C21orf140	0.001317903	UpRegulated
RGPD6	0.001369672	UpRegulated
NKAIN3	0.001547773	DownRegulated
LINC01314	0.001724048	DownRegulated
RNA5-8SP6	0.00179445	DownRegulated
LOC102724669	0.001815741	UpRegulated
SCARNA21	0.001901803	DownRegulated
LOC105376900	0.001967423	UpRegulated
FOLH1	0.002045369	DownRegulated
CERCAM	0.002073422	DownRegulated
WNT7A	0.002106385	UpRegulated
CHGA	0.002266758	UpRegulated
RNY1	0.002311453	DownRegulated
MT1G	0.002974524	UpRegulated
KCNQ5-IT1	0.003036445	UpRegulated
SNORD3D	0.003227766	UpRegulated
TBR1	0.003415595	UpRegulated
LOC101927424	0.003476867	DownRegulated
VSTM2A	0.003503165	UpRegulated
TTR	0.00350484	DownRegulated
GPC5-AS2	0.004173881	DownRegulated
SPDYE9P	0.004307481	DownRegulated
SNORD114-14	0.004412503	UpRegulated
SPTSSB	0.004912232	UpRegulated
CNP	0.005039642	DownRegulated
SNORD116-20	0.00522652	UpRegulated
SNORD116-3	0.005447324	UpRegulated
SNORD116-9	0.005447324	UpRegulated
WDR86	0.005815413	DownRegulated
CTNNA3	0.007487912	DownRegulated
TF	0.007660946	DownRegulated

INA	0.007948262	UpRegulated
SNORA43	0.008405193	UpRegulated
RPL31P62	0.008877157	UpRegulated
KCNC1	0.009732848	UpRegulated
FAM149A	0.010162961	DownRegulated
KL	0.013159034	DownRegulated
SNORD116-15	0.014468649	UpRegulated
SOSTDC1	0.015072577	UpRegulated
ANGPTL4	0.016433699	DownRegulated
MGST1	0.016576884	DownRegulated
COL4A5	0.016978396	DownRegulated
LOC400927	0.018375736	UpRegulated
DPY19L3	0.023526594	DownRegulated
PLN	0.024303641	UpRegulated
SPARC	0.026302852	DownRegulated
TRNM	0.027427947	UpRegulated
ZNF726P1	0.027512444	UpRegulated
PDGFRA	0.02836967	DownRegulated
LOC105376832	0.030603608	UpRegulated
CHI3L2	0.032299415	DownRegulated
KCNA2	0.03435468	UpRegulated
SNHG5	0.034520887	DownRegulated
LOC105374713	0.034737234	DownRegulated
ADAMTS18	0.035896245	DownRegulated
DEAR	0.03847978	UpRegulated
RBM3	0.039322856	DownRegulated
LOC105369154	0.040108011	UpRegulated
MAG	0.043533163	DownRegulated
BEST1	0.044239352	DownRegulated
LOC100506725	0.04652215	DownRegulated
HIST3H2A	0.047788744	UpRegulated
SNORD116-22	0.048950777	UpRegulated
SMOC1	0.049754958	DownRegulated

Supplemental Table 3: The 93 differentially expressed genes using samples from HIP and TCx brain regions sorted using P-values.

Gene Symbol	Corrected Sorted Pvalue	Status in AD
FIBCD1	8.71E-16	DownRegulated
RNVU1-7	1.29E-13	DownRegulated
RNU6-33P	7.14E-13	UpRegulated
MTCO1P2	7.45E-13	DownRegulated
RPS23P8	7.89E-12	DownRegulated

LOC102723728	5.22E-10	DownRegulated
RNY1	7.13E-10	DownRegulated
LOC400927-CSNK1E	1.36E-09	DownRegulated
RPL15P3	1.63E-08	UpRegulated
CABP7	3.12E-08	DownRegulated
LOC101929800	3.72E-08	DownRegulated
SLC14A1	1.06E-07	DownRegulated
RNVU1-10	1.20E-07	UpRegulated
SCARNA21	1.24E-07	DownRegulated
ANKRD20A19P	2.05E-07	DownRegulated
C1QTNF9B	5.55E-07	DownRegulated
LOC642423	5.88E-07	UpRegulated
TRNV	7.60E-07	UpRegulated
ADIRF	9.28E-07	DownRegulated
LOC105379032	2.95E-06	DownRegulated
LOC440896	4.42E-06	DownRegulated
IL12RB2	5.41E-06	DownRegulated
RNA5-8SP6	1.14E-05	DownRegulated
ANGPTL4	1.62E-05	DownRegulated
FNDC1	2.10E-05	DownRegulated
TRNP	2.21E-05	DownRegulated
HIST1H4K	2.42E-05	UpRegulated
OPALIN	2.89E-05	DownRegulated
RPL31P62	3.07E-05	UpRegulated
PVALB	5.05E-05	UpRegulated
CCL3	5.11E-05	UpRegulated
LOC105379510	5.29E-05	UpRegulated
PMS2P8	7.38E-05	DownRegulated
LYPD1	7.66E-05	DownRegulated
FPGT-TNNI3K	0.000100581	DownRegulated
LOC105379509	0.000130687	UpRegulated
LINC01314	0.000132551	DownRegulated
LOC102724669	0.000157449	UpRegulated
SNORD116-15	0.000186476	DownRegulated
NKX6-2	0.000195327	UpRegulated
FAM183A	0.000219707	DownRegulated
LOC105374713	0.00034894	DownRegulated
VGF	0.000351342	DownRegulated
LOC105370750	0.000420712	UpRegulated
LOC105379481	0.00066243	DownRegulated
IFIT1	0.000828579	UpRegulated
C1QL2	0.00133756	DownRegulated

WDR86	0.001900843	DownRegulated
LOC105379480	0.002527909	UpRegulated
ISG15	0.002675067	UpRegulated
WASH5P	0.00269743	UpRegulated
LOC100421372	0.002809966	DownRegulated
NPTX2	0.002941024	DownRegulated
SNORD116-8	0.003787569	UpRegulated
TXNIP	0.006021709	DownRegulated
PDGFRA	0.006290504	DownRegulated
SNORD116-17	0.006403845	UpRegulated
SNORD116-19	0.006403845	UpRegulated
TRNE	0.006832345	UpRegulated
KL	0.007073099	DownRegulated
CD38	0.007174611	DownRegulated
RGS10	0.00733464	DownRegulated
MIR4441	0.007688668	DownRegulated
LOC101927424	0.008587228	DownRegulated
OR2A9P	0.009684459	DownRegulated
SPTLC1P1	0.009878683	UpRegulated
ETNPPL	0.012121424	DownRegulated
LOC105369154	0.012390047	UpRegulated
SERTAD4	0.012559172	UpRegulated
SNORD116-3	0.013270905	UpRegulated
SNORD116-9	0.013270905	UpRegulated
HBB	0.016329132	DownRegulated
SNORA2A	0.016768354	DownRegulated
LOC100420440	0.017723732	UpRegulated
LOC101929179	0.018664273	DownRegulated
F3	0.019072295	DownRegulated
LOC105377901	0.019257512	DownRegulated
LOC105369390	0.019967426	DownRegulated
GADD45A	0.02036427	DownRegulated
RNA5-8SP3	0.02077953	DownRegulated
SNORA67	0.020933933	DownRegulated
EMP1	0.021629736	DownRegulated
COX3	0.022587612	DownRegulated
TRNM	0.024211821	UpRegulated
GJB6	0.027388139	DownRegulated
S100A16	0.030030869	DownRegulated
RNA18SP2	0.03343635	UpRegulated
SYT2	0.033567919	UpRegulated
DUSP6	0.037139848	DownRegulated

SNORA75	0.040709091	DownRegulated
LOC102724604	0.042179133	DownRegulated
SNORD8	0.042491405	UpRegulated
EIF4EP2	0.049587044	DownRegulated

Supplemental Table 4: Gene Ontology Biological Processes Annotations using all 602 DEGs obtained from the four brain region analysis.

Analysis Type: PANTHER Overrepresentation Test (Released 20200407)

Annotation Version and Release Date: GO Ontology database DOI: 10.5281/zenodo.3727280 Released 2020-03-23

**GO biological process complete**	**P-value**
nervous system development (GO:0007399)	8.00E-36
synaptic signaling (GO:0099536)	3.83E-33
chemical synaptic transmission (GO:0007268)	2.74E-32
anterograde trans-synaptic signaling (GO:0098916)	2.74E-32
trans-synaptic signaling (GO:0099537)	4.92E-32
regulation of biological quality (GO:0065008)	6.19E-23
cell-cell signaling (GO:0007267)	2.32E-22
regulation of transport (GO:0051049)	7.08E-22
system development (GO:0048731)	3.53E-21
generation of neurons (GO:0048699)	5.25E-21
neurogenesis (GO:0022008)	6.21E-21
modulation of chemical synaptic transmission (GO:0050804)	3.07E-20
regulation of trans-synaptic signaling (GO:0099177)	3.39E-20
regulation of ion transport (GO:0043269)	2.51E-19
regulation of localization (GO:0032879)	6.94E-19
regulation of membrane potential (GO:0042391)	4.38E-18
multicellular organism development (GO:0007275)	5.01E-18
synapse organization (GO:0050808)	2.03E-17
cell junction organization (GO:0034330)	7.69E-17
anatomical structure development (GO:0048856)	3.84E-16
multicellular organismal process (GO:0032501)	2.52E-15
developmental process (GO:0032502)	7.89E-15
regulation of nervous system development (GO:0051960)	8.10E-15
regulation of ion transmembrane transport (GO:0034765)	8.30E-15
signaling (GO:0023052)	1.34E-14
vesicle-mediated transport in synapse (GO:0099003)	1.62E-14
metal ion transport (GO:0030001)	2.20E-14
synaptic vesicle cycle (GO:0099504)	2.23E-14
inorganic ion transmembrane transport (GO:0098660)	2.40E-14

regulation of transmembrane transport (GO:0034762)	6.40E-14
neuron development (GO:0048666)	2.08E-13
cell communication (GO:0007154)	2.30E-13
neuron differentiation (GO:0030182)	3.06E-13
ion transport (GO:0006811)	8.47E-13
neurotransmitter transport (GO:0006836)	3.17E-12
potassium ion transport (GO:0006813)	3.58E-12
regulation of neuron projection development (GO:0010975)	5.11E-12
potassium ion transmembrane transport (GO:0071805)	5.99E-12
regulation of neurogenesis (GO:0050767)	1.71E-11
neurotransmitter secretion (GO:0007269)	1.79E-11
signal release from synapse (GO:0099643)	1.79E-11
regulation of neuron differentiation (GO:0045664)	3.52E-11
inorganic cation transmembrane transport (GO:0098662)	5.99E-11
ion transmembrane transport (GO:0034220)	6.29E-11
cation transport (GO:0006812)	6.69E-11
regulation of synaptic plasticity (GO:0048167)	7.46E-11
regulation of synapse structure or activity (GO:0050803)	9.99E-11
localization (GO:0051179)	1.22E-10
regulation of multicellular organismal process (GO:0051239)	1.62E-10
regulation of cell development (GO:0060284)	2.46E-10
transport (GO:0006810)	3.12E-10
regulation of neurotransmitter levels (GO:0001505)	3.54E-10
central nervous system development (GO:0007417)	3.92E-10
cation transmembrane transport (GO:0098655)	8.22E-10
establishment of localization (GO:0051234)	9.10E-10
regulation of plasma membrane bounded cell projection organization (GO:0120035)	1.28E-09
regulation of cell projection organization (GO:0031344)	2.08E-09
transmembrane transport (GO:0055085)	2.79E-09
monovalent inorganic cation transport (GO:0015672)	3.48E-09
regulation of cell morphogenesis (GO:0022604)	3.86E-09
synaptic vesicle exocytosis (GO:0016079)	3.98E-09
export from cell (GO:0140352)	9.04E-09
regulation of exocytosis (GO:0017157)	1.35E-08
cell differentiation (GO:0030154)	3.30E-08
regulation of cell morphogenesis involved in differentiation (GO:0010769)	7.23E-08
secretion by cell (GO:0032940)	8.77E-08
regulation of synaptic vesicle cycle (GO:0098693)	9.60E-08
cellular developmental process (GO:0048869)	1.01E-07
neuron projection development (GO:0031175)	1.02E-07
regulation of synapse organization (GO:0050807)	1.37E-07

exocytosis (GO:0006887)	1.53E-07
cellular process (GO:0009987)	1.65E-07
behavior (GO:0007610)	1.70E-07
regulation of neurotransmitter secretion (GO:0046928)	2.13E-07
cell development (GO:0048468)	2.46E-07
regulation of multicellular organismal development (GO:2000026)	2.87E-07
calcium-ion regulated exocytosis (GO:0017156)	3.19E-07
regulation of neuronal synaptic plasticity (GO:0048168)	3.36E-07
regulation of system process (GO:0044057)	3.71E-07
axon development (GO:0061564)	3.74E-07
secretion (GO:0046903)	4.62E-07
signal release (GO:0023061)	6.15E-07
positive regulation of nervous system development (GO:0051962)	6.20E-07
regulation of developmental process (GO:0050793)	6.87E-07
regulation of neurotransmitter transport (GO:0051588)	7.93E-07
regulation of vesicle-mediated transport (GO:0060627)	2.05E-06
regulation of cell differentiation (GO:0045595)	2.11E-06
regulation of cellular localization (GO:0060341)	2.61E-06
regulation of secretion (GO:0051046)	3.57E-06
regulation of cation transmembrane transport (GO:1904062)	4.00E-06
regulation of regulated secretory pathway (GO:1903305)	4.11E-06
regulation of amine transport (GO:0051952)	8.38E-06
regulation of secretion by cell (GO:1903530)	8.92E-06
neuron projection morphogenesis (GO:0048812)	9.59E-06
regulation of synaptic vesicle exocytosis (GO:2000300)	1.01E-05
regulation of signaling (GO:0023051)	1.02E-05
plasma membrane bounded cell projection morphogenesis (GO:0120039)	1.18E-05
learning or memory (GO:0007611)	1.18E-05
cell projection morphogenesis (GO:0048858)	1.45E-05
cellular component organization (GO:0016043)	2.03E-05
biological regulation (GO:0065007)	2.17E-05
regulation of metal ion transport (GO:0010959)	2.25E-05
positive regulation of synaptic transmission (GO:0050806)	2.28E-05
regulation of cellular component organization (GO:0051128)	2.33E-05
axonogenesis (GO:0007409)	2.78E-05
regulation of cellular process (GO:0050794)	3.09E-05
positive regulation of neuron differentiation (GO:0045666)	3.13E-05
regulation of postsynaptic membrane potential (GO:0060078)	3.23E-05
multicellular organismal signaling (GO:0035637)	3.50E-05
cell part morphogenesis (GO:0032990)	4.36E-05
regulation of axonogenesis (GO:0050770)	5.37E-05
cognition (GO:0050890)	5.60E-05

regulation of cell communication (GO:0010646)	5.68E-05
plasma membrane bounded cell projection organization (GO:0120036)	6.28E-05
head development (GO:0060322)	6.33E-05
cell morphogenesis involved in neuron differentiation (GO:0048667)	8.72E-05
brain development (GO:0007420)	1.05E-04
regulated exocytosis (GO:0045055)	1.27E-04
positive regulation of transport (GO:0051050)	1.64E-04
regulation of biological process (GO:0050789)	1.73E-04
regulation of anatomical structure morphogenesis (GO:0022603)	1.78E-04
regulation of cation channel activity (GO:2001257)	1.94E-04
calcium ion-regulated exocytosis of neurotransmitter (GO:0048791)	2.16E-04
cell projection organization (GO:0030030)	2.33E-04
cell morphogenesis (GO:0000902)	2.46E-04
cellular component morphogenesis (GO:0032989)	2.67E-04
cellular component organization or biogenesis (GO:0071840)	2.68E-04
nucleic acid metabolic process (GO:0090304)	3.07E-04
regulation of transporter activity (GO:0032409)	3.27E-04
positive regulation of neuron projection development (GO:0010976)	3.27E-04
positive regulation of multicellular organismal process (GO:0051240)	4.19E-04
regulation of developmental growth (GO:0048638)	4.76E-04
negative regulation of cell morphogenesis involved in differentiation (GO:0010771)	5.91E-04
nucleobase-containing compound metabolic process (GO:0006139)	6.29E-04
positive regulation of neurogenesis (GO:0050769)	7.11E-04
synapse assembly (GO:0007416)	7.48E-04
regulation of catecholamine secretion (GO:0050433)	8.69E-04
negative regulation of neuron projection development (GO:0010977)	8.92E-04
heterocycle metabolic process (GO:0046483)	1.04E-03
protein localization to synapse (GO:0035418)	1.19E-03
regulation of ion transmembrane transporter activity (GO:0032412)	1.21E-03
positive regulation of exocytosis (GO:0045921)	1.37E-03
biological_process (GO:0008150)	1.68E-03
regulation of dopamine secretion (GO:0014059)	1.72E-03
cellular nitrogen compound metabolic process (GO:0034641)	2.01E-03
regulation of transmembrane transporter activity (GO:0022898)	2.11E-03
neural nucleus development (GO:0048857)	2.17E-03
negative regulation of cell projection organization (GO:0031345)	2.21E-03
action potential (GO:0001508)	2.47E-03
regulation of nervous system process (GO:0031644)	2.51E-03
transmission of nerve impulse (GO:0019226)	2.54E-03
organic cyclic compound metabolic process (GO:1901360)	2.92E-03
nervous system process (GO:0050877)	3.05E-03
vesicle-mediated transport (GO:0016192)	3.26E-03

learning (GO:0007612)	3.27E-03
cell junction assembly (GO:0034329)	3.48E-03
regulation of cell size (GO:0008361)	3.50E-03
negative regulation of cell development (GO:0010721)	3.74E-03
response to calcium ion (GO:0051592)	4.22E-03
exocytic process (GO:0140029)	4.35E-03
regulation of calcium ion-dependent exocytosis (GO:0017158)	4.42E-03
synaptic vesicle recycling (GO:0036465)	4.76E-03
positive regulation of developmental process (GO:0051094)	4.97E-03
protein localization to cell junction (GO:1902414)	5.14E-03
establishment of localization in cell (GO:0051649)	5.14E-03
positive regulation of cell development (GO:0010720)	6.08E-03
regulation of dendrite development (GO:0050773)	6.92E-03
cellular aromatic compound metabolic process (GO:0006725)	7.05E-03
positive regulation of ion transport (GO:0043270)	8.38E-03
presynaptic endocytosis (GO:0140238)	1.03E-02
synaptic vesicle endocytosis (GO:0048488)	1.03E-02
associative learning (GO:0008306)	1.06E-02
positive regulation of cellular component organization (GO:0051130)	1.06E-02
vesicle docking (GO:0048278)	1.13E-02
regulation of dendrite morphogenesis (GO:0048814)	1.40E-02
membrane depolarization (GO:0051899)	1.41E-02
neuromuscular process (GO:0050905)	1.45E-02
system process (GO:0003008)	1.46E-02
positive regulation of calcium ion-dependent exocytosis (GO:0045956)	1.91E-02
positive regulation of nervous system process (GO:0031646)	1.91E-02
regulation of response to drug (GO:2001023)	2.11E-02
cell morphogenesis involved in differentiation (GO:0000904)	2.23E-02
protein localization to cell periphery (GO:1990778)	2.24E-02
positive regulation of cell projection organization (GO:0031346)	2.31E-02
cellular response to calcium ion (GO:0071277)	2.38E-02
glutamate secretion (GO:0014047)	2.41E-02
vesicle docking involved in exocytosis (GO:0006904)	2.56E-02
anatomical structure morphogenesis (GO:0009653)	2.60E-02
chloride transport (GO:0006821)	3.12E-02
axon guidance (GO:0007411)	3.59E-02
regulation of heart contraction (GO:0008016)	3.59E-02
cellular response to stimulus (GO:0051716)	3.72E-02
memory (GO:0007613)	3.91E-02
cellular localization (GO:0051641)	4.01E-02
substantia nigra development (GO:0021762)	4.15E-02
neuron projection guidance (GO:0097485)	4.22E-02

negative regulation of neuron differentiation (GO:0045665)	4.43E-02
negative regulation of nervous system development (GO:0051961)	4.50E-02
regulation of synapse assembly (GO:0051963)	4.55E-02
negative regulation of developmental growth (GO:0048640)	4.99E-02

Supplemental Table 5: Gene Ontology Molecular Functions Annotations using all 602 DEGs obtained from the four brain region analysis.

Analysis Type: PANTHER Overrepresentation Test (Released 20200407)

Annotation Version and Release Date: GO Ontology database DOI: 10.5281/zenodo.3727280 Released 2020-03-23

**GO molecular function complete**	**P-value**
gated channel activity (GO:0022836)	6.18E-21
voltage-gated ion channel activity (GO:0005244)	1.08E-17
voltage-gated channel activity (GO:0022832)	1.26E-17
ion channel activity (GO:0005216)	3.67E-17
channel activity (GO:0015267)	6.92E-17
passive transmembrane transporter activity (GO:0022803)	7.54E-17
voltage-gated cation channel activity (GO:0022843)	2.08E-16
voltage-gated potassium channel activity (GO:0005249)	3.38E-14
metal ion transmembrane transporter activity (GO:0046873)	8.44E-14
cation channel activity (GO:0005261)	2.65E-13
potassium channel activity (GO:0005267)	3.46E-13
potassium ion transmembrane transporter activity (GO:0015079)	1.86E-12
inorganic molecular entity transmembrane transporter activity (GO:0015318)	4.14E-11
transporter activity (GO:0005215)	5.00E-11
transmembrane transporter activity (GO:0022857)	3.25E-10
calmodulin binding (GO:0005516)	4.03E-10
ion transmembrane transporter activity (GO:0015075)	5.70E-10
inorganic cation transmembrane transporter activity (GO:0022890)	6.50E-10
monovalent inorganic cation transmembrane transporter activity (GO:0015077)	2.52E-09
cation transmembrane transporter activity (GO:0008324)	6.54E-09
syntaxin-1 binding (GO:0017075)	1.38E-07
SNARE binding (GO:0000149)	6.82E-07
syntaxin binding (GO:0019905)	1.21E-06
calcium ion binding (GO:0005509)	5.57E-06
delayed rectifier potassium channel activity (GO:0005251)	1.05E-05
neurotransmitter receptor activity (GO:0030594)	4.78E-05
ligand-gated channel activity (GO:0022834)	8.06E-05
ligand-gated ion channel activity (GO:0015276)	8.06E-05
nucleic acid binding (GO:0003676)	1.23E-04
lipid binding (GO:0008289)	2.02E-04
structural constituent of myelin sheath (GO:0019911)	2.55E-04
ligand-gated anion channel activity (GO:0099095)	4.73E-04
phospholipid binding (GO:0005543)	6.06E-04
calcium-dependent phospholipid binding (GO:0005544)	7.89E-04
postsynaptic neurotransmitter receptor activity (GO:0098960)	3.30E-03
GABA receptor activity (GO:0016917)	1.00E-02

neurotransmitter receptor activity involved in regulation of postsynaptic membrane potential (GO:0099529)	1.04E-02
voltage-gated sodium channel activity (GO:0005248)	1.28E-02
protein kinase binding (GO:0019901)	1.34E-02
anion binding (GO:0043168)	1.49E-02
extracellular ligand-gated ion channel activity (GO:0005230)	1.74E-02
transmitter-gated channel activity (GO:0022835)	1.77E-02
transmitter-gated ion channel activity (GO:0022824)	1.77E-02
cytoskeletal protein binding (GO:0008092)	2.30E-02
inhibitory extracellular ligand-gated ion channel activity (GO:0005237)	2.36E-02
protein binding (GO:0005515)	2.59E-02

Supplemental Table 6: Gene Ontology Cellular Components Annotations using all 602 DEGs obtained from the four brain region analysis.

Analysis Type: PANTHER Overrepresentation Test (Released 20200407)

Annotation Version and Release Date: GO Ontology database DOI: 10.5281/zenodo.3727280 Released 2020-03-23

**GO cellular component complete**	**P-value**
synapse (GO:0045202)	2.64E-69
cell junction (GO:0030054)	1.42E-61
neuron projection (GO:0043005)	1.89E-41
postsynapse (GO:0098794)	7.68E-37
presynapse (GO:0098793)	1.25E-33
axon (GO:0030424)	4.15E-32
synaptic membrane (GO:0097060)	2.23E-31
somatodendritic compartment (GO:0036477)	4.19E-30
plasma membrane bounded cell projection (GO:0120025)	1.53E-28
glutamatergic synapse (GO:0098978)	6.18E-28
cell projection (GO:0042995)	7.62E-28
plasma membrane region (GO:0098590)	7.02E-24
cell periphery (GO:0071944)	7.48E-24
postsynaptic membrane (GO:0045211)	2.50E-23
plasma membrane (GO:0005886)	9.62E-23
neuron to neuron synapse (GO:0098984)	6.36E-21
neuronal cell body (GO:0043025)	1.10E-19
dendritic tree (GO:0097447)	1.26E-19
postsynaptic specialization (GO:0099572)	1.41E-19
cell body (GO:0044297)	4.31E-19
dendrite (GO:0030425)	4.98E-19
integral component of synaptic membrane (GO:0099699)	5.37E-19
intrinsic component of synaptic membrane (GO:0099240)	6.42E-19
postsynaptic density (GO:0014069)	1.61E-18
asymmetric synapse (GO:0032279)	3.09E-18

presynaptic membrane (GO:0042734)	3.81E-18
intrinsic component of plasma membrane (GO:0031226)	7.74E-18
integral component of plasma membrane (GO:0005887)	2.58E-17
membrane (GO:0016020)	3.01E-17
ion channel complex (GO:0034702)	4.82E-16
transmembrane transporter complex (GO:1902495)	1.01E-15
transporter complex (GO:1990351)	2.29E-15
distal axon (GO:0150034)	5.23E-15
intrinsic component of postsynaptic membrane (GO:0098936)	5.29E-14
integral component of postsynaptic membrane (GO:0099055)	1.52E-13
GABA-ergic synapse (GO:0098982)	1.50E-12
cation channel complex (GO:0034703)	2.46E-12
Schaffer collateral - CA1 synapse (GO:0098685)	8.40E-12
transport vesicle (GO:0030133)	1.32E-11
voltage-gated potassium channel complex (GO:0008076)	9.32E-11
intrinsic component of membrane (GO:0031224)	1.04E-10
cellular_component (GO:0005575)	1.27E-10
perikaryon (GO:0043204)	1.57E-10
exocytic vesicle membrane (GO:0099501)	1.60E-10
synaptic vesicle membrane (GO:0030672)	1.60E-10
postsynaptic specialization membrane (GO:0099634)	2.55E-10
main axon (GO:0044304)	3.07E-10
cellular anatomical entity (GO:0110165)	4.53E-10
intrinsic component of presynaptic membrane (GO:0098889)	4.62E-10
neuron projection terminus (GO:0044306)	4.65E-10
exocytic vesicle (GO:0070382)	6.84E-10
potassium channel complex (GO:0034705)	6.92E-10
integral component of presynaptic membrane (GO:0099056)	7.06E-10
integral component of membrane (GO:0016021)	9.42E-10
transport vesicle membrane (GO:0030658)	1.32E-09
postsynaptic density membrane (GO:0098839)	1.60E-09
synaptic vesicle (GO:0008021)	3.47E-09
axon terminus (GO:0043679)	1.06E-08
intrinsic component of postsynaptic specialization membrane (GO:0098948)	2.31E-08
integral component of postsynaptic specialization membrane (GO:0099060)	9.37E-08
intrinsic component of postsynaptic density membrane (GO:0099146)	1.61E-07
plasma membrane protein complex (GO:0098797)	3.88E-07
integral component of postsynaptic density membrane (GO:0099061)	6.58E-07
cytoplasmic vesicle (GO:0031410)	1.93E-06
intracellular vesicle (GO:0097708)	2.86E-06
growth cone (GO:0030426)	6.14E-06
site of polarized growth (GO:0030427)	1.23E-05

intrinsic component of synaptic vesicle membrane (GO:0098563)	1.66E-05
vesicle (GO:0031982)	2.16E-05
neuron projection membrane (GO:0032589)	2.52E-05
cytoplasmic vesicle membrane (GO:0030659)	3.56E-05
presynaptic active zone (GO:0048786)	4.04E-05
dendritic spine (GO:0043197)	4.44E-05
neuron spine (GO:0044309)	5.28E-05
calyx of Held (GO:0044305)	6.45E-05
secretory vesicle (GO:0099503)	8.04E-05
vesicle membrane (GO:0012506)	1.10E-04
membrane protein complex (GO:0098796)	2.00E-04
leading edge membrane (GO:0031256)	2.23E-04
whole membrane (GO:0098805)	2.91E-04
excitatory synapse (GO:0060076)	3.51E-04
dendrite membrane (GO:0032590)	5.00E-04
nucleus (GO:0005634)	5.35E-04
integral component of synaptic vesicle membrane (GO:0030285)	9.45E-04
endomembrane system (GO:0012505)	1.16E-03
dense core granule (GO:0031045)	1.28E-03
bounding membrane of organelle (GO:0098588)	1.57E-03
cell leading edge (GO:0031252)	1.65E-03
neuronal cell body membrane (GO:0032809)	2.05E-03
axon initial segment (GO:0043194)	2.34E-03
cell body membrane (GO:0044298)	3.92E-03
GABA receptor complex (GO:1902710)	6.68E-03
node of Ranvier (GO:0033268)	9.17E-03
neuromuscular junction (GO:0031594)	1.14E-02
chloride channel complex (GO:0034707)	1.23E-02
sarcolemma (GO:0042383)	1.33E-02
cell-cell junction (GO:0005911)	1.56E-02
voltage-gated sodium channel complex (GO:0001518)	1.64E-02
catalytic complex (GO:1902494)	1.87E-02
